# Insights into the Stearoyl-Acyl Carrier Protein Desaturase (SAD) Family in Tigernut (*Cyperus esculentus* L.), an Oil-Bearing Tuber Plant

**DOI:** 10.3390/plants14040584

**Published:** 2025-02-14

**Authors:** Zhi Zou, Xiaowen Fu, Chunqiang Li, Xiaoping Yi, Jiaquan Huang, Yongguo Zhao

**Affiliations:** 1 National Key Laboratory for Tropical Crop Breeding, Hainan Key Laboratory for Biosafety Monitoring and Molecular Breeding in Off-Season Reproduction Regions, Institute of Tropical Biosciences and Biotechnology/Sanya Research Institute of Chinese Academy of Tropical Agricultural Sciences, Haikou 571101, China; xiaowen9924@126.com (X.F.); lichunqiang@itbb.org.cn (C.L.); yixiaoping@itbb.org.cn (X.Y.); 2 School of Breeding and Multiplication, Sanya Institute of Breeding and Multiplication, College of Tropical Agriculture and Forestry, Hainan University, Sanya 572025, China; 3 College of Biology and Food Engineering, Guangdong University of Petrochemical Technology, Maoming 525000, China

**Keywords:** vegetative tissue, underground tuber, oleic acid, FAB2, AAD, structural variation, expression divergence

## Abstract

Plant oils rich in oleic acid (OA) are attracting considerable attention for their high nutritional value and significant industrial potential. Stearoyl-acyl carrier protein desaturases (SADs) are a class of soluble desaturases that play a key role in OA accumulation in plants. In this study, the first genome-wide characterization of the *SAD* gene family was conducted in tigernut (*Cyperus esculentus* L. var. *sativus* Baeck., Cyperaceae), an oil-rich tuber plant typical for its high OA content. Six *SAD* genes identified from the tigernut genome are comparative to seven reported in two model plants *Arabidopsis thaliana* and *Oryza sativa*, but relatively more than four were found in most Cyperaceae species examined in this study. A comparison of 161 *SAD* genes from 29 representative plant species reveals the monogenic origin and lineage-specific family evolution in Poales. *C. esculentus SAD* genes (*CeSADs*) were shown to constitute two evolutionary groups (i.e., FAB2 and AAD) and four out of 12 orthogroups identified in this study, i.e., FAB2a, FAB2b, FAB2c, and AAD1. Whereas FAB2a and AAD1 are widely distributed, FAB2b and FAB2c are specific to Cyperaceae, which may arise from FAB2a via tandem and dispersed duplications, respectively. Though FAB2d and AAD2 are also broadly present in monocots, they are more likely to be lost in the Cyperaceae ancestor sometime after the split with its close family, Juncaceae. In tigernut, FAB2a appears to have undergone species-specific expansion via tandem duplication. Frequent structural variation and apparent expression divergence were also observed. Though FAB2a and AAD1 usually feature two and one intron, respectively, gain of certain introns was observed in *CeSAD* genes, all of which have three introns. Despite recent expansion of the FAB2 group, *CeFAB2-1* has evolved into the dominant member that was highly and constitutively expressed in all tested organs. Moreover, *CeFAB2-1*, *CeAAD1,* as well as *CeFAB2-5* have evolved to be predominantly expressed in tubers and thus contribute to high OA accumulation. These findings highlight lineage-specific evolution of the *SAD* family and putative roles of *CeSAD* genes in tuber oil accumulation, which facilitate further functional analysis and genetic improvement in tigernut and other species.

## 1. Introduction

Lipids in the form of triacylglycerols (TAGs) are the major energy and carbon reserve that are not only essential for plant development but also widely used for food, fuel, and biomaterials [1]. TAGs usually accumulate in the seeds of oil crops, e.g., soybean (*Glycine max*), peanut (*Arachis hypogaea*), rapeseed (*Brassica napus*), sesame (*Sesamum indicum*), sunflower (*Helianthus annuus*), safflower (*Carthamus tinctorius*), maize (*Zea mays*), and oil tea (*Camellia oleifera*) [2]. Whereas the majority of them belong to the eudicot clade, monocots, especially cereal crops, which provide us food and/or industrial materials, are generally low in oil content. For example, TAG accumulation in maize is mostly confined to the embryo and scutellum, which occupy less than 10% of the kernel weight [3]. TAGs also accumulate in some vegetative organs such as underground tubers. A good example for high oil accumulation (up to 35%) in tubers is tigernut (*Cyperus esculentus* L. var. *sativus* Baeck.), a Cyperaceae plant within the Poales order of the monocot clade [4,5,6,7]. Like oil tea, tigernut oil is typical for the high oleic acid (OA, 18:1) content (67.7–74.6%), which is attracting considerable attention for its high nutritional value and significant industrial potential [8,9,10]. However, the underlying mechanism is poorly understood.

Stearoyl-acyl carrier protein (ACP) desaturases (SADs) are a class of soluble desaturases specific to photosynthetic organisms, which are located in the stroma of chloroplasts [1]. They could introduce a double bond at the Δ9 position of fatty acids (FA) bounded to ACP, e.g., palmitic acid (PA, 16:0) and stearic acid (SA, 18:0) [11,12,13,14]. Since SADs are the only known enzymes catalyzing the conversion of 18:0 to 18:1 in plants, their activity primarily determines the ratio of saturated to monounsaturated FAs (MUFAs) and plays a key role in 18:1 accumulation [15,16,17]. In plants, SADs are encoded by a small family and there are seven members present in arabidopsis (*Arabidopsis thaliana*), i.e., *FATTY ACID BIOSYNTHESIS 2* (*FAB2*)/*SUPPRESSOR OF SA INSENSITIVE 2* (*SSI2*) and *ACYL-ACP DESATURASE 1–6* (*AtAAD1–6*). Enzymatic analysis showed that all AtSADs possess both 18:0 and 16:0-ACP Δ9 desaturase activity, though AtAAD1–6 exhibits greatly reduced specificity toward 18:0-ACP [16]. Among four members (i.e., *AtFAB2*, *AtAAD5*, *AtAAD1*, and *AtAAD6*) that contribute to the 18:1 pool in seeds, *AtFAB2* is predominant and its mutant *ssi2* accumulates increased levels of 18:0 and reduced levels of 18:1 [18,19]. Moreover, the native expression levels of six *AtAAD* genes could not complement the loss-of-function mutation in *ssi2* [16]. On the contrary, ω-7 FA production in the *A. thaliana* aleurone was mainly contributed by AtAAD2 and -3, two members also called palmitoyl-ACP desaturase (PAD) [20]. Recently, two SAD-coding genes named *CeSAD1* and *-2* were also isolated from tigernut tubers, whose expression patterns during tuber development were positively correlated with FA and oil accumulation dynamics. Moreover, their overexpression in yeast (*Saccharomyces cerevisiae*) and tobacco (*Nicotiana tabacum*) supported that these two CeSADs had high desaturase activity to catalyze MUFA biosynthesis [21]. Nevertheless, a genome-wide characterization of *CeSAD* genes has not been reported yet. The recently available tigernut genome [22] provides an opportunity to address this issue.

In this study, a number of six *SAD* genes were identified from the tigernut genome, which could be assigned into two evolutionary groups named FAB2 and AAD as observed in *A. thaliana*. Interestingly, though the amounts of the *SAD* family are comparative between tigernut and *A. thaliana*, the group composition is highly different and extensive gene expansion mainly via local duplication was found for FAB2 and AAD, respectively. The origin and evolutionary patterns of *CeSAD* genes were further investigated via comparison with those identified from 28 representative plant species, which reveals lineage and/or species-specific evolution. Herein, we report our findings.

## 2. Results

### 2.1. Characterization of Six SAD Genes in Tigernut

**Figure 1:**
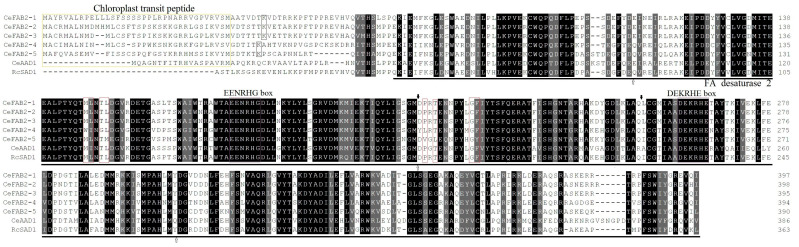
Multiple sequence alignment of CeSAD proteins with structure-resolved RcSAD1. Sequence alignment was conducted using MUSCLE, where the mature peptide of RcSAD1 is under the NCBI accession number of 1OQ4_A. Identical and similar amino acids are highlighted in black or dark grey, respectively. The positions of the phase 2 intron in *CeFAB2-1–5* are boxed in black, whereas the phase 0 introns are indicated using solid (*CeFAB2-1–5*) and hollow (*CeAAD1*) arrows (see more in Figure S1). The conserved FA_desaturase_2 domain is underlined, and several typical structural features are boxed in different colors, i.e., chloroplast transit peptide (gold), two histidine boxes (i.e., EENRHG and DEKRHE) (purple), and key residues determining substrate specificity and double-bond position (red) (AAD: acyl-ACP desaturase; Ce: *C. esculentus*; FA: fatty acid; FAB2: fatty acid biosynthesis 2; Rc: *R. communis*; SAD: stearoyl-ACP desaturase).

As shown in Table 1, a total of six SAD-coding genes were identified from the tigernut genome, and all of their deduced peptides possess one FA_desaturase_2 domain (under the Pfam accession number of PF03405) that is specific to this family. The protein length varies from 386 to 398 amino acids (AAs) with a molecular weight (MW) of 43.72–45.78 kilodalton (kDa), whereas the grand average of hydropathicity (GRAVY) values are less than 0 (from −0.435 to −0.496) (Table 1), implying their hydrophilic feature. Except for CeFAB2-4, others harbor the theoretical isoelectric point (pI) values of less than 7.00 (5.87–6.34) (Table 1), implying their basic feature. Multiple sequence alignment indicates that the short protein length and low MW of CeAAD1 are due to a short N-terminal region relative to other members, though all of them contain several typical structural features, i.e., chloroplast transit peptide and two histidine boxes (i.e., EENRHG and DEKRHE). Notably, AA substitutions were observed at key residues determining substrate specificity and double-bond position (Figure 1), which correspond to M^114^, L^115^, T^117^, L^118^, P^179^, T^181^, G^188^, and F^189^ of the mature peptide of *Ricinus communis* SAD1 (RcSAD1) [11], implying possible functional divergence.

**Figure 2:**
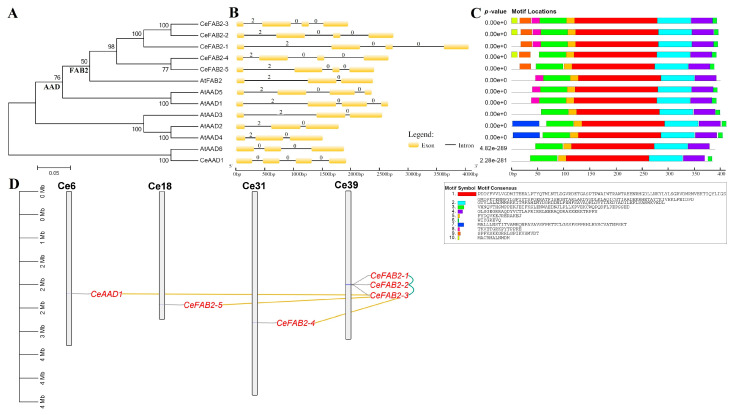
Chromosomal localization, duplication events, and structural and phylogenetic analyses of *CeSAD* genes. (**A**) Shown is an unrooted phylogenetic tree resulting from full-length Ce/AtSAD proteins with MEGA6 (maximum likelihood method and bootstrap of 1000 replicates), where the distance scale denotes the number of amino-acid substitutions per site. The name of each clade is indicated next to the corresponding group. (**B**) Shown are the exon-intron structures, where the numbers indicate intron phases. Whereas 0 indicates the intron that is located between codons, 2 indicates the intron that is located between the second and third bases of a codon. (**C**) Shown is the distribution of conserved motifs among Ce/AtSAD proteins, where different motifs are represented by different color blocks, as indicated, and the same color block in different proteins indicates a certain motif. (**D**) Shown are chromosomal localization and duplication events of *CeSAD* genes, where dispersed and tandem repeats are connected using gold and blue lines, respectively (AAD: acyl-ACP desaturase; At: *A. thaliana*; Ce: *C. esculentus*; FA: fatty acid; FAB2: fatty acid biosynthesis 2; Mb: Megabase; SAD: stearoyl-ACP desaturase; Scf: scaffold).

To disclose the evolutionary relationships, an unrooted phylogenetic tree was constructed using full-length proteins of *SAD* genes present in tigernut and *A. thaliana*. As shown in Figure 2A, two main groups were identified. For convenience, they were named FAB2-like and AAD-like. Interestingly, the FAB2 group includes AtFAB2 and five tigernut members (i.e., CeFAB2-1–5), whereas the AAD group contains CeAAD1 and six *A. thaliana* members (i.e., AtAAD1–6), implying species or lineage-specific expansion. Correspondingly, AtFAB2 exhibits 74.52–85.01% sequence similarities with five CeFAB2s, relatively higher than 72.09% with CeAAD1, whereas CeAAD1 shares 74.63% similarity with AtAAD6, relatively higher than 69.69–72.57% with other AtAADs (Table S1). Interestingly, gene structure analysis reveals that all *CeSAD* genes possess four exons and three introns. By contrast, only *AtAAD1* and *-3* harbor three introns, whereas others contain two introns (Figure 2B). Since all *SAD* genes in the basal angiosperm *Amborella trichopoda* [23] feature two introns (see below), species or lineage-specific gain of the third intron (all in phase 0 that is located between codons) in *CeSAD* genes (Figure 1, Figure 2B and Figure S1) could be speculated. Notably, in contrast to most members featuring phase 2 (where the intron is located between the second and third bases of a codon) for the first intron, a phase 0 intron with a distinct position was observed in *CeAAD1* and *AtAAD6* (Figure 1, Figure 2B and Figure S1), implying different origins. Moreover, the position of the third intron of *CeAAD1* is similar to *AtAAD1* and *AtAAD5*, but different from that of five *CeFAB2s* (Figure S1), supporting independent evolution. In accordance with sequence alignment, conserved motif analysis using MEME shows that SADs are highly conserved, which usually possess nine out of ten motifs identified in this study, i.e., Motifs 10, 9, 8, 3, 5, 1, 2, 4, and 6. Nevertheless, the sequence-specific loss of certain motifs was also observed. Motif 10, which was characterized as the chloroplast signal peptide, was only retained in CeFAB2-2, -3, and -4, and was placed by Motif 7 in AtAAD2 and -4, implying high sequence divergence. Whereas Motif 9 is limited to five CeFAB2s, Motif 8 is only present in CeFAB2-1, CeFAB2-2, CeFAB2-3, AtFAB2, AtAAD1, and AtAAD5. Additionally, the widely distributed Motif 6 is absent from AtFAB2 and AtAAD6 (Figure 2C).

Further gene localization shows that six *CeSAD* genes are distributed over four scaffolds (Scfs), i.e., Scf6, Scf18, Scf31, and Scf39 (Table 1). Whereas Scf39 includes three members (*CeFAB2-1*, *-2*, and *-3*) and was characterized as the hotspot for tandem duplication, others possess a single one (Figure 2D). Similar cases were also observed for *A. thaliana*, where Chr3 (*AtAAD2*, *-4*, and *-5*) and Chr5 (*AtAAD1* and *-3*) are the hotspots for tandem duplication (Figure S2). Moreover, duplication event analysis indicates that *CeFAB2-3*–*5* and *CeAAD1* are dispersed repeats (Figure 2D), whereas *AtAAD2* and *-3* were characterized as whole-genome duplication (WGD) and dispersed repeats of *AtAAD3* and *-6*, respectively (Figure S2).

### 2.2. Comparison of SAD Genes from Representative Plant Species Reveals Lineage-Specific Family Evolution in Poales

To uncover the origin and evolution of *CeSAD* genes, homologs were further identified from 28 representative plant species, which resulted in 154 members. Notably, despite the presence of four members in *A. trichopoda*, all of them belong to FAB2 and harbor two introns in phases 2 and 0 (Figure S3), implying the late origin of AAD. By contrast, one, two, and two *AAD* genes were identified from eelgrass (*Zostera marina*), duckweed (*Spirodela polyrhiza*), and *Acorus gramineus*, three early diverged monocots that did not experience the τ WGD shared by all core monocots [24,25,26]. Unlike *AtrSAD* genes, both *AgAAD1* and *SpAAD1* possess a single intron, whereas *ZmaAAD1* is intronless, in contrast to the presence of two introns in *SpAAD2* and *AgAAD2* (Table S2), implying early divergence into two AAD subgroups during subsequent evolution.

**Figure 3:**
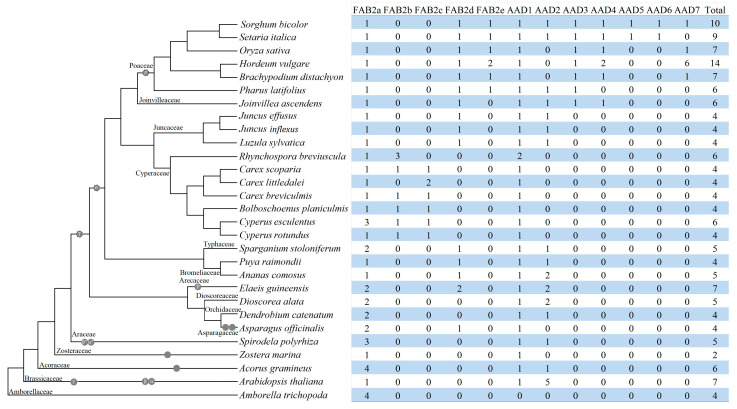
Species-specific distribution of 12 orthogroups in 29 representative plant species. The species tree is referred to NCBI Taxonomy (https://www.ncbi.nlm.nih.gov/taxonomy, accessed on 20 August 2024) and recent WGDs or triplications resulting in polyploidy (CoGepedia; https://genomevolution.org/coge/, accessed on 20 August 2024) are marked. Names of tested plant families are indicated next to the corresponding branches (AAD: acyl-ACP desaturase; FAB2: fatty acid biosynthesis 2; WGD: whole-genome duplication).

To gain insights into the lineage-specific evolution of *SAD* genes, orthologs among different species were identified using Orthofinder. As shown in Figure 3, a total of 12 orthogroups were obtained and six *CeSAD* genes belong to FAB2a, FAB2b, FAB2c, and AAD1, respectively. Significantly, *CeFAB2-1*, *-2*, and *-3* all belong to the widely distributed FAB2a that also includes four *AtrSAD* genes (Figure 3), implying tigernut-specific expansion via tandem duplication. By contrast, FAB2b and FAB2c were only found in Cyperaceae species (Figure 3), implying their birth sometime after the split with the close family Juncaceae. Since FAB2a and FAB2b members in *Bolboschoenus planiculmis*, *Rhynchospora breviuscula*, *Carex breviculmis*, and *C. scoparia* are organized in tandem repeats (Table S2), this means that FAB2b may arise from FAB2a via Cyperaceae-specific tandem duplication followed by chromosomal rearrangement and gene loss in tigernut and *C. littledalei*. Except for the absence from *R. breviuscula*, FAB2c was characterized as the dispersed repeat of FAB2a in other Cyperaceae species examined in this study (Table S2). FAB2d is present in most Poales species beyond Cyperaceae (Figure 3), implying lineage-specific contraction. Interestingly, like FAB2a, FAB2d members in *Juncus inflexus* (*JiFAB2-2*) and *J. effusus* (*JeFAB2-2*) also possess four introns in phases 2, 0, 0, and 1 (located between the first and second bases of a codon), which is different from phases 2, 0, 0, and 0 as observed in *JiFAB2-1* and *JeFAB2-1*. By contrast, their ortholog in *Luzula sylvatica* (*LsFAB2-2*) was shown to have three introns in phases 2, 0, and 1 (Figure S3). Additionally, FAB2e is confined to Poaceae species, implying lineage-specific expansion. Among seven AAD groups identified in this study, AAD1 and -2 were widely present, though the latter was lost in all tested Cyperaceae species. AAD3 and -4 were found in *Joinvillea ascendens* and most Poaceae species examined, though AAD4 was absent from *Pharus latifolius*. On the contrary, AAD5–7 was limited to Poaceae species, though species-specific expansion and contraction were observed (Figure 3). Notably, though AAD2 usually features two introns in phases 2 and 0, both *JiAAD2* and *JeAAD2* were shown to have gained one additional phase 1 intron, whereas *LsAAD2* has two introns in phases 1 and 0, implying Juncaceae-specific gain of the second intron (phase 1) followed by species-specific loss of the first intron (phase 2) in *LsAAD2* (Figure S3).

**Figure 4:**
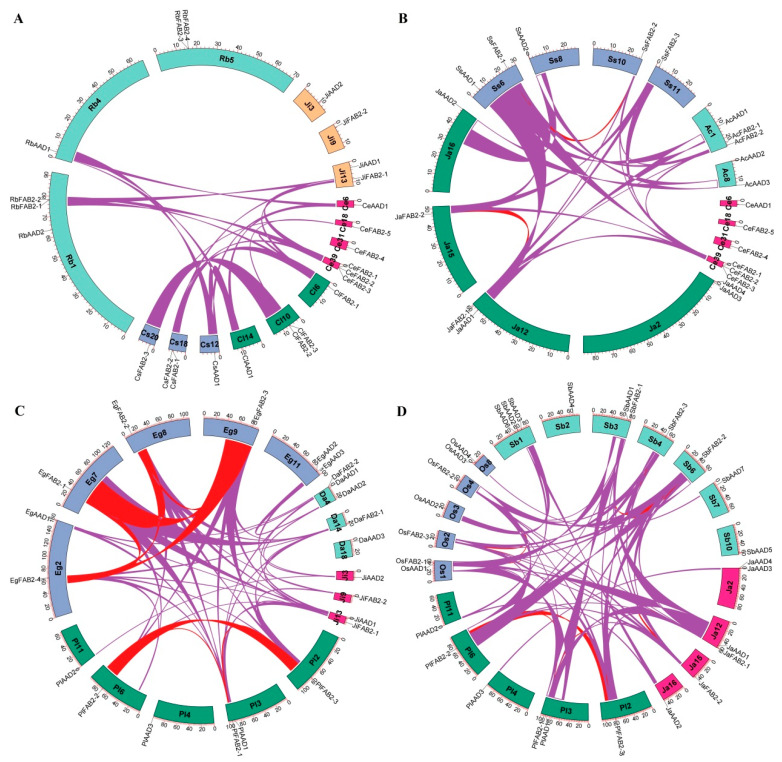
Synteny analysis within and between *C. esculentus* and representative plant species. (**A**) Synteny analysis within and between *C. esculentus*, *C. littledalei*, *C. scoparia*, *R. breviuscula*, and *J. inflexus*. (**B**) Synteny analysis within and between *C. esculentus*, *J. ascendens*, *S. stoloniferum*, and *A. comosus*. (**C**) Synteny analysis within and between *J. inflexus*, *P. latifolius*, *E. guineensis*, and *D. alata*. (**D**) Synteny analysis within and between *J. ascendens*, *P. latifolius*, *O. sativa*, and *S. bicolor*. Shown are *SAD* gene-encoding chromosomes/scaffolds and only syntenic blocks containing *SAD* genes are marked, where red and purple lines indicate intra- and inter-species, respectively. The scale is in Mb (AAD: acyl-ACP desaturase; Ac: *A. comosus*; Ce: *C. esculentus*; Cl: *C. littledalei*; Cs: *C. scoparia*; Da: *D. alata*; Eg: *E. guineensis*; FAB2: fatty acid biosynthesis 2; Ja: *J. ascendens*; Ji: *J. inflexus*; Mb: megabase; Os: *O. sativa*; Pl: *P. latifolius*; Rb: *R. breviuscula*; Sb: *S. bicolor*; Ss: *S. stoloniferum*).

Further interspecific synteny analyses reveal that three *CeSAD* genes, i.e., *CeFAB2-1*, *CeFAB2-5*, and *CeAAD1*, have syntelogs in at least 1 out of 28 representative species examined in this study. Whereas syntelogs for *CeFAB2-5* and *CeAAD1* were only found in Cyperaceae species (Figure 4A), *CeFAB2-1* syntelogs are also present in all other Poales species tested (Figure 4B,C) as well as oil palm (*Elaeis guineensis*) (Figure 4C), *Dioscorea alata* (Figure 4C), garden asparagus (*Asparagus officinalis*), and apostasia (*Dendrobium catenatum*). Interestingly, despite the close biological relationship between Juncaceae and Cyperaceae, three Juncaceae species, *J. inflexus*, *J. effusus*, and *L. sylvatica*, exhibit a distinct evolution pattern from Cyperaceae species, which include FAB2a, FAB2d, AAD1, and AAD2, and only the FAB2a member *JiFAB2-1* is still located within syntenic blocks with that of Cyperaceae species (Figure 4A, where *J. inflexus* has been presented as an example for one of the Juncaceae species). By contrast, all *JiSAD* genes were shown to harbor syntelogs in *P. latifolius*, *E. guineensis*, and *D. alata* (Figure 4C), supporting the early divergence of these four orthogroups. Though *JiFAB2-1* and *-2* are no longer located within syntenic blocks, both of them were characterized as syntelogs of *DaFAB2-1*, *EgFAB2-1*/*-2*/*-3*/*-4*, and *PlFAB2-1*/*-2*/*-3* (Figure 4C), providing direct evidence of WGD derivation of FAB2d from FAB2a, most likely via the τ WGD [27]. In *E. guineensis* and *P. latifolius*, further expansion was contributed by p and ρ WGDs that are specific to the Arecaceae and Poaceae families, respectively [24]. Compared with Poales species, *JaAAD3* (a dispersed repeat of *JaAAD2*) also has a syntelog in *P. latifolius*, but not any other Poaceae species examined (Figure 4D, where *O. sativa* and *S. bicolor* were shown as two representatives in Poaceae), implying possible chromosome rearrangement before Poaceae radiation.

### 2.3. Organ-Specific Transcriptome Profiling Reveals Expression Divergence of CeSAD Genes

**Figure 5:**
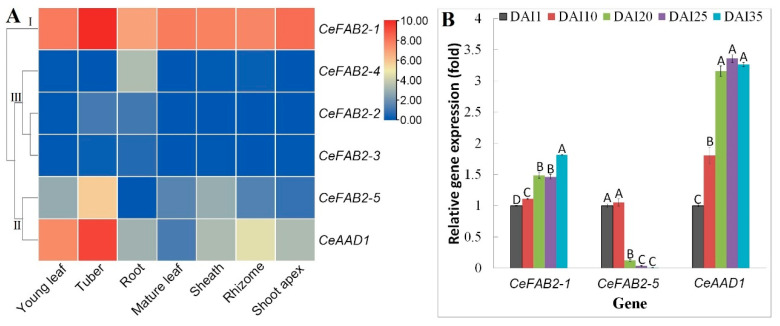
Expression profiles of *CeSAD* genes. (**A**) Organ-specific expression profiles of six *CeSAD* genes. The heatmap was generated using the R package implemented with a row-based standardization. Color scale represents FPKM normalized log_2_ transformed counts, where blue indicates low expression and red indicates high expression. (**B**) Expression profiles of *CeFAB2-1*, *CeFAB2-5*, and *CeAAD1* at different stages of tuber development. All values for three tested genes at DAI1 were normalized to 1, whereas bars indicate SD (N = 3) and uppercase letters indicate difference significance tested following Duncan’s one-way multiple-range post hoc ANOVA (*p* < 0.01) (AAD: acyl-ACP desaturase; Ce: *C. esculentus*; DAI: days after tuber initiation; FAB2: fatty acid biosynthesis 2; FPKM: Fragments per kilobase of exon per million fragments mapped).

To provide a global view of expression profiles of *CeSAD* genes, transcriptomes of seven main organs, i.e., shoot apex, young leaf, mature leaf, sheath, root, rhizome, and tuber, were examined. As shown in Figure 5A, distinct expression patterns were observed, and transcripts were detected most in tubers, followed by young leaves, shoot apexes, mature leaves, sheaths, and rhizomes, and least in roots. Whereas *CeFAB2-2*, *-3*, and *-4* were lowly expressed in all tested samples (with the FPKM value less than 1), *CeFAB2-1* and *CeAAD1* have evolved to be constitutively expressed and *CeFAB2-5* was preferentially expressed in tubers. Moreover, *CeFAB2-1* transcripts were relatively more than those of *CeAAD1* in all samples examined, contributing to more than 85% of total *CeSAD* transcripts in shoot apexes, mature leaves, leaf sheaths, rhizomes, and roots. In young leaves, *CeFAB2-1* and *CeAAD1* contributed to 57% and 42% of the total transcripts, respectively, whereas in tubers, *CeFAB2-1*, *CeAAD1*, and *CeFAB2-5* contributed to 59%, 38%, and 3%, respectively. Based on their expression patterns, six *CeSAD* genes were clustered into three groups: Group I included *CeFAB2-1* that was highly abundant in all tested samples; Group II contained *CeFAB2-5* and *CeAAD1*; and Group III included three barely expressed members, i.e., *CeFAB2-2*, *-3*, and *-4* (Figure 5A). These results support the expression and possible functional divergence of *CeSAD* genes and especially between *CeFAB2-1* and its paralogs *CeFAB2-2* and *-3*).

### 2.4. Expression Profiles of CeSAD Genes During Tuber Development

To learn more about the expression profiles of *CeSAD* genes during tuber development, three tuber-expressed members, i.e., *CeFAB2-1*, *CeFAB2-5*, and *CeAAD1*, were selected for qRT-PCR analysis on the basis of the above transcriptome profiling, and the five tested stages were 1, 10, 20, 25, and 35 days after tuber initiation (DAI), representing initiation, early swelling, middle swelling, late swelling, and maturation, respectively [28]. As shown in Figure 5B, gradual upregulation during tuber development was observed for *CeFAB2-1* and *CeAAD1*, which peaked at DAI35 and DAI25, respectively. For *CeFAB2-1*, transcript abundance between DAI20 and DAI25 was not significant, whereas no significant difference was detected for the expression level of *CeAAD1* at three later stages. By contrast, a gradual transcript decrease was observed for *CeFAB2-5*, though no significant difference was found between two early (10 vs. 1 DAI) and two later stages (35 vs. 25 DAI) (Figure 5B).

## 3. Discussion

### 3.1. The Tigernut Genome Contains a High Number of Six SAD Genes, and the Family Expansion Was Contributed by Tandem and Dispersed Duplications

The importance of *SAD* genes in 18:1 accumulation has prompted us to study this special gene family in tigernut, a Cyperaceae plant producing high levels of oil with up to 75% 18:1 in underground tubers [4,10,28]. Six family members identified from the tigernut genome are comparative to seven present in two model plants *A. thaliana* and rice (*Oryza sativa*) [16], but relatively more than four found in most Cyperaceae species, e.g., *C. rotundus*, *C. littledalei*, *C. scoparia*, *C. breviculmis*, and *B. planiculmis*, though the amounts identified from the *C. rotundus* transcriptome assembly [29] may be under estimated. Among them, only *CeFAB2-1* (*CeSAD1*) and *CeAAD1* (*CeSAD2*) have been described before [21]. According to phylogenetic analysis, six *CeSAD* genes could be assigned into two main groups as described in *A. thaliana*, i.e., FAB2 (5) and AAD (1). Interestingly, the group composition is highly different from *A. thaliana*, which contains one FAB2 and six AADs [16], implying lineage or species-specific evolution. Correspondingly, our comparative genomic analysis reveals the roles of WGD, tandem, dispersed, and transposed duplications on the expansion of *SAD* genes (more precisely is the AAD group) in *A. thaliana* (Figure S2), whereas the family expansion in tigernut (more precisely is the FAB2 group) was contributed by tandem and dispersed duplications, which is also different from Poaceae species where extensive expansion was observed in both groups (Figure 3).

### 3.2. Comparative Genomics Analysis Reveals the Monogenic Origin and Lineage-Specific Evolution of the SAD Family in Poales

To gain insights into the origin and evolution of *CeSAD* genes, a total of 161 family members identified from 29 representative plant species were included for comparison. These species belong to 15 plant families (Figure 3). Among them, *A. trichopoda* represents the basal angiosperm that did not experience any recent WGD [23]. By contrast, the model eudicot *A. thaliana* has been proven to undergo three WGDs (γ, β, and α) after the split with *A. trichopoda*, whereas most monocots except for orders Acorales and Alismatales shared the τ WGD [23,24,25,30], and Poaceae species represented by *O. sativa* further underwent the Poales-specific σ WGD as well as the Poaceae-specific ρ WGD [27]. Orthologous analysis on the basis of these species resulted in 12 orthogroups, i.e., FAB2a, FAB2b, FAB2c, FAB2d, FAB2e, and AAD1–7 (Figure 3). Presence of the sole FAB2a in *A. trichopoda* implies the monogenic origin of this gene family in angiosperms, which rapidly diverged into FAB2a, AAD1, and AAD2 as early as in early diverged monocots, e.g., *A. gramineus* and duckweed [24,26]. In contrast to a role of the Brassicaceae-specific α WGD on the expansion of AAD2 in *A. thaliana* (Figure S2), nearly half of the monocots examined in this study were shown to preserve two FAB2 repeats derived from the τ WGD, i.e., FAB2a and FAB2d, though the latter seems to be absent from the whole Cyperaceae family (Figure 3). Moreover, a role of recent WGDs on FAB2 expansion was also observed in a high number of monocots, e.g., *A. gramineus* [26], *A. officinalis* [31], *E. guineensis* (p WGD) [32], *Sparganium stoloniferum* [33], and the majority of Poaceae species (ρ WGD) examined in this study. By contrast, despite the extensive expansion of AAD in Poaceae, none of them were characterized as WGD repeats, supporting lineage-specific evolution that is different from *A. thaliana* (Table S2). Interestingly, though AAD2 is widely distributed, it was lost in all Cyperaceae species examined in this study (Figure 3), implying lineage-specific contraction, which may occur sometime after the Cyperaceae-Juncaceae split. Notably, Cyperaceae is the good example of FAB2 expansion via tandem duplication, which resulted in FAB2b sometime before the radiation of the plant family. Moreover, further species-specific expansion via tandem duplication was also observed in tigernut, *C. littledalei*, and *R. breviuscula* (Table S2). Similar cases were also reported for plasma membrane intrinsic protein (PIP)-coding genes [34], which may contribute to species adaptation [35]. In summary, possible evolution routes of the *SAD* family in angiosperms are as follows: the family first diverged into FAB2a, AAD1, and AAD2 before the monocot–eudicot split; in the model eudicot plant *A. thaliana*, AAD2 was further expanded via the Brassicaceae-specific α WGD followed by tandem duplication; in the monocot clade, FAB2a first gave rise to FAB2d along with the τ WGD, and FAB2d further generated FAB2e via the Poaceae-specific ρ WGD; in Cyperaceae, whereas both FAB2d and AAD2 were lost, FAB2a was expanded via tandem and dispersed duplications; in Joinvilleaceae and Poaceae, AAD2 first gave birth to AAD3 and -4 followed by AAD5–7, mainly via dispersed duplication.

In addition to gene copies, lineage-specific gain and loss of certain introns in different phases were also observed. While AAD1 features a single intron in phase 0, FAB2s and AAD2 usually possess two introns in phases 2 and 0, respectively. However, all AAD1 members in Cyperaceae and Juncaceae have gained two additional phase 0 introns as observed in *CeAAD1* (Figure 2B and Figure S3), implying that the event may have occurred sometime before the Cyperaceae-Juncaceae divergence but after the split with other Poales species. Similarly, for FAB2a, the last common ancestor of Cyperaceae and Juncaceae first gained one phase 0 intron as observed in CeFAB2s, followed by gain of one additional phase 0 intron in Juncaceae species, e.g., *LsFAB2-1*, *JeFAB2-1*, and *JiFAB2-1*. As for FAB2d, *LsFAB2-2* gained one phase 1 intron, whereas *JeFAB2-2* and *JiFAB2-2* gained one phase 1 intron as well as one additional phase 0 intron. However, we are not sure about the time of gaining these two introns. One possible model is that the Juncaceae ancestor gained two introns followed by species-specific loss of the phase 0 intron in *L. sylvatica*, whereas another model is that the Juncaceae ancestor first gained the phase 0 intron followed by *Juncus*-specific gain of the phase 1 intron. Sequencing more genomes in this genus may provide more information. Interestingly, the position of the phase 0 intron found in *LsFAB2-2*, *JeFAB2-2*, and *JiFAB2-2* is similar to the first intron as observed in *CeAAD1*, implying that this site may be the hotspot for intron insertion. Cyperaceae- and Juncaceae-specific gain of certain introns were also described in the *oleosin* gene family [29], though the biological significance has not been clarified.

### 3.3. CeSAD Genes Underwent Apparent Expression Divergence

Expression profiles of *CeSAD* genes were further studied, which reveals apparent expression divergence. Despite recent expansion of the FAB2 group after the split with the Juncaceae family, i.e., Cyperaceae-specific expansion for *CeFAB2-4* and *-5* and species-specific expansion for *CeFAB2-1*–*3* (Figure 3), *CeFAB2-1* has evolved into the dominant member that was highly and constitutively expressed in all organs examined in this study; this is in contrast to *CeFAB2-2*–*4*, which were lowly expressed. *CeAAD1* was also shown to be constitutively expressed, though its abundance is lower than that of *CeFAB2-1* (Figure 5). Moreover, *CeFAB2-1*, *CeAAD1*, as well as *CeFAB2-5* all exhibit a tuber-preferential expression pattern, co-expressing with genes associated with FA biosynthesis, as observed in oil-bearing seeds [16,17,36], which is in accordance with significant accumulation of oil and 18:1 in this special tissue [29]. Similar expression patterns for *CeFAB2-1* and *CeAAD1* were also reported by Li and colleagues [21]. Since both CeFAB2-1 and CeAAD1 have been proven to harbor high desaturase activity toward 18:0-ACP [21], they could be the main contributors to high 18:1 accumulation but traces of 16:1 in tigernut tubers [36]. Unlike CeFAB2-1 and CeAAD1, two *A. thaliana* members, i.e., AtAAD2 and -3, have evolved to possess a substrate toward 16:0-ACP, and contribute to the ω-7 FA production in the aleurone [14,20]. Notably, this neofunctionalization is more likely to be independent among different species, and evidences are as follows: firstly, *AtAAD2* and *-3* are very young repeats that were derived from the Brassicaceae-specific α WGD [30], and their tandem repeats, i.e., *AtAAD4/-5* and *-1*, respectively, prefer a substrate toward 18:0-ACP [16]; secondly, only a few plant species could accumulate significant ω-7 monounsaturated FAs, e.g., macadamia nuts (*Macadamia sp.*), cat’s claw vine (*Doxantha unguiscati*), and sea buckthorn (*Hippophae rhamnoides*) [37,38,39]. Sequence alignment, homology modeling, and site-directed mutagenesis experiments suggested that the F residue corresponding to T^181^ of RcSAD1 [11] plays a key role in determining the substrate specificity in AtAAD2 and AtAAD3 [20]. In tigernut, no such substitution was observed in all six CeSADs, which may explain why no detectable ω-7 FAs have been found in this species [9,10,21,36].

## 4. Conclusions

This is the first genome-wide analysis of the *SAD* gene family in tigernut, an oil-rich tuber plant in Cyperaceae. A high number of six *CeSAD* genes were identified, which represent two evolutionary groups and 5 out of 12 defined orthogroups. Identification and comparison of 161 members from 29 plant species representing 15 plant families support the monogenic origin of this gene family in angiosperms and lineage-specific evolution in Poales. In contrast to the extensive expansion of both FAB2 and AAD in Poaceae, the Cyperaceae ancestor is more likely to harbor only two members (one FAB2a and one AAD1) and the expansion of the FAB2 group was contributed by tandem and dispersed duplications. Apparent expression divergence was also observed, where *CeFAB2-1*, *CeAAD1*, as well as *CeFAB2-5* have evolved to be predominantly expressed in tubers and thus contribute to the high 18:1 accumulation in this special tissue. These findings facilitate further functional analysis and genetic improvement in tigernut and other species.

## 5. Materials and Methods

### 5.1. Identification of SAD Family Genes

Genome sequences of representative plant species were obtained from Phytozome v13 (https://phytozome.jgi.doe.gov/pz/portal.html, accessed on 20 August 2024), NCBI (https://www.ncbi.nlm.nih.gov/, accessed on 20 August 2024), Genome Warehouse (https://ngdc.cncb.ac.cn/gwh/, accessed on 20 August 2024), TAIR11 (https://www.arabidopsis.org/, accessed on 20 August 2024), and RGAP 7 (https://rice.uga.edu/, accessed on 20 August 2024): *A. trichopoda* (v2.1), *A. thaliana* (Araport11), *A. gramineus* (v1), *Z. marina* (v3.1), *S. polyrhiza* (v2), *A. officinalis* (v1.1), *D. catenatum* (v1), *D. alata* (v1), *E. guineensis* (v3), *Ananas comosus* (v3), *Puya raimondii* (v1), *S. stoloniferum* (v1), *J. ascendens* (v1), *P. latifolius* (v1), *O. sativa* (v7.0), *Hordeum vulgare* (Morex V3), *Brachypodium distachyon* (v3.2), *Setaria italica* (v2.2), *Sorghum bicolor* (v5.1), *L. sylvatica* (v1), *J. effusus* (v1), *J. inflexus* (v1), *B. planiculmis* (v1), *R. breviuscula* (v1), *C. breviculmis* (v1), *C. scoparia* (v1), *C. littledalei* (v1), and *C. esculentus* (v1). As for *C. rotundus* whose genome sequences are not available, the de novo assembled tuber transcriptome as described before [29] was used. To identify *SAD* family genes, HMMER (v3.3.2, http://hmmer.janelia.org/, accessed on 20 August 2024) searches were conducted using the Pfam profile PF03405 (v35.0, https://pfam.xfam.org/, accessed on 20 August 2024). Gene models of candidates were manually revised as previously described [40], whereas gene structures were displayed using GSDS 2.0 (https://gsds.gao-lab.org/, accessed on 20 August 2024). The presence of the conserved FA_desaturase_2 domain in deduced proteins was confirmed using Pfam Search (http://pfam.xfam.org/, accessed on 20 August 2024), and biochemical parameters were calculated using ProtParam (http://web.expasy.org/protparam/, accessed on 20 August 2024).

### 5.2. Phylogenetic and Conserved Motif Analyses

Multiple sequence alignments of deduced proteins were performed using MUSCLE implemented in MEGA6 [41], and phylogenetic tree construction was performed using MEGA6 with the maximum likelihood method and bootstrap of 1000 replicates. Conserved motifs were identified using MEME (v5.4.1, https://meme-suite.org/tools/meme, accessed on 20 August 2024) with the following parameters: any number of repetitions; maximum number of motifs, 10; and the optimum width of each motif, between 5 and 250 residues.

### 5.3. Synteny Analysis and Definition of Orthogroups

Synteny analysis was carried out as described before [42]. Gene duplication modes were identified using the DupGen_finder pipeline [35], and orthologous genes were clustered using Orthofinder (v2.3.8) [43].

### 5.4. Gene Expression Analysis Based on RNA-Seq

Global expression profiles of *CeSAD* genes were analyzed using the Illumina RNA-seq dataset under the NCBI BioProject accession number of PRJNA703731, which includes seven main organs (i.e., stem apex, young leaf, mature leaf, sheath of mature leaf, root, rhizome, and tuber) that were collected 85 days after sowing (DAS). Quality control of raw reads and subsequent read mapping were carried out as described before [40], and relative gene expression level was presented as fragments per kilobase of exon per million fragments mapped (FPKM) [44].

### 5.5. Gene Expression Analysis Based on qRT-PCR

In this experiment, a tigernut variety named Reyan3 [8] was employed, and plants were grown as previously described [29,45]. The whole growth period was approximately 85 d and new tubers were initiated at the apex of the stolons at about 30 days after sowing. Five representative developing tubers with three biological replicates were collected at 1, 10, 20, 25, and 35 days after tuber initiation. Total RNA extraction, synthesis of the first-strand cDNA, and qRT-PCR analysis were conducted as described before [5]. Primers used in this study are shown in Table S3, where *CeTIP41* and *CeUCE2* were employed as two reference genes as previously described [5]. The relative expression levels of target genes were normalized using the mean of the reference genes.

## Figures and Tables

**Table 1:** *SAD* genes identified in *C. esculentus* (AA: amino acid; AAD: acyl-ACP desaturase; Ce: *C. esculentus*; FA: fatty acid; FAB2: fatty acid biosynthesis 2; GRAVY: grand average of hydropathicity; kDa: kilodalton; MW: molecular weight; pI: isoelectric point; Scf: scaffold).

Gene Name	Locus ID	Position	AA	MW (kDa)	pI	GRAVY	FA_desaturase_2
*CeFAB2-1*	CESC_22416	Scf39:2000859 2004917(+)	397	45.54	5.87	−0.459	69..390
*CeFAB2-2*	CESC_22415	Scf39:2008494 2011485(−)	398	45.78	5.89	−0.496	68..391
*CeFAB2-3*	CESC_22414	Scf39:2016213 2018165(+)	395	45.37	6.00	−0.486	66..388
*CeFAB2-4*	CESC_23282	Scf31:2811812 2814471(+)	394	45.06	7.08	−0.435	65..387
*CeFAB2-5*	CESC_22943	Scf18:2428057 2430442(−)	390	44.93	6.34	−0.440	59..383
*CeAAD1*	CESC_12146	Scf6:2194845 2196771(+)	386	43.72	6.10	−0.467	48..380

## Data Availability

Transcriptome data used in this study are under the NCBI accession number of PRJNA703731.

## Supplementary Materials

The following supporting information can be downloaded at: https://www.mdpi.com/article/10.3390/plants14040584/s1, Figure S1: Gene models for *Ce/AtSAD* genes (AAD: acyl-ACP desaturase; At: *A. thaliana*; Ce: *C. esculentus*; FAB2: fatty acid biosynthesis 2; SAD: stearoyl-ACP desaturase). Figure S2: Chromosomal localization and duplication events of *AtSAD* family genes, where WGD, tandem, dispersed, and transposed repeats are connected using red, blue, gold, and purple lines, respectively (AAD: acyl-ACP desaturase; At: *A. thaliana*; FAB2: fatty acid biosynthesis 2; Mb: Megabase; SAD: stearoyl-ACP desaturase). Figure S3: Gene structures of *SAD* family genes in *A. trichopoda*, *J. effuses*, *J. inflexus*, and *L. sylvatica* (AAD: acyl-ACP desaturase; Atr: *A. trichopoda*; CDS: coding sequence; FAB2: fatty acid biosynthesis 2; Je: *J. effusus*; Ji: *J. inflexus*; kb: kilobase; Ls: *L. sylvatica*; SAD: stearoyl-ACP desaturase). Table S1: Percent similarity within and between CeSADs and AtSADs (AAD: acyl-ACP desaturase; At: *A. thaliana*; Ce: *C. esculentus*; FAB2: fatty acid biosynthesis 2; SAD: stearoyl-ACP desaturase). **Table S2**: *SAD* family genes identified in representative plant species (AAD: acyl-ACP desaturase; Ac: *A. comosus*; Ag: *A. gramineus*; Ao: *Asparagus officinalis*; At: *A. thaliana*; Atr: *A. trichopoda*; Bd: *B. distachyon*; Bp: *B. planiculmis*; Cb: *C. breviculmis*; Chr: chromosome; Cl: *C. littledalei*; Cr: *C. rotundus*; Cs: *C. scoparia*; Eg: *E. guineensis*; Hv: *H. vulgare*; Ja: *J. ascendens*; Je: *J. effusus*; Ji: *J. inflexus*; Ls: *L. sylvatica*; Pl: *P. latifolius*; Pr: *P. raimondii*; Rb: *R. breviuscula*; Scf: scaffold; Sb: *S. bicolor*; Si: *S. italica*; Sp: *S. polyrhiza*; Ss: *S. stoloniferum*; WGD: whole-genome duplication). **Table S3**: Primers used in this study (AAD: acyl-ACP desaturase; Ce: *C. esculentus*; FAB2: fatty acid biosynthesis 2).
