# Supplementary material for: Insights into the Stearoyl-Acyl Carrier Protein Desaturase (SAD) Family in Tigernut (Cyperus esculentus L.), an Oil-Bearing Tuber Plant
Source: Plants (Basel). 2025 Feb 14;14(4):584. doi: 10.3390/plants14040584 (PMC11859870; doi:10.3390/plants14040584)
Supplement: Supplementary file 1 [file plants-14-00584-s001.zip › Figure S1.pdf]

Figure S1. Gene models for *Ce/AtSAD* genes. (**A**) *CeFAB2-1*. (**B**) *CeFAB2-2*. (**C**) *CeFAB2-3*. (**D**) *CeFAB2-4*. (**E**) *CeFAB2-5*. (**F**) *CeAAD1*. (**G**) *AtFAB2*. (**H**) *AtAAD1*. (**I**) *AtAAD2*. (**J**) *AtAAD3*. (**K**) *AtAAD4*. (**L**) *AtAAD5*. (**M**) *AtAAD6*. The coding region is marked with uppercase letters, under which are the deduced amino acids. The start and stop codons are marked with bold letters, whereas the intron region is marked with lowercase letters.

(**A**) *CeFAB2-1*

1 M A Y R V A L R P E L L L S F S S S S P
1 **ATG**GCGTACCGAGTGGCGCTGCGGCCGGAGCTCTTACTCTCCTTCTCCTCCTCCTCCCCC
21 P L R P N A R R V G P V R V S M A A T V
61 CCGCTGCGGCCCAATGCGCGCCGCGTGGGCCCCGTCCGCGTCTCCATGGCCGCCACTGTC
41 D T K
121 GACACCAAgtgagctccctttctttctttttgtttttttttctctgtttggaatctcgt
181 agatacgaatcgattggtgaattcgtcgattgacgaatgctgctgctgctgttaatatct
241 gaatttgttatttttgggtttggtttggtggggaaggcgtttttttgggatctctgggtct
301 gggggtttatcgattgattgcggagatggcgactgcaattcgagggtttttgattgattg
361 attgattgattgggtcacgccgttttgggggttttgtaagaaatcagcttccgcatgcat
421 caattcgtcactcgtttcgcatgaatggtgctttttttaaggaagaaatattcaataaaat
481 aaaataaattaaaataaattaaattgttaaataaattaaaatttatctttcgtcatgagt
541 cttaatgagtgctacaagtgaagtgagtgaggctctatgttgaattgttgtgttgatcgg
601 ttcaaattactgactatttccatcagatttcccttgattctgtgaattgggggtaattgc
661 cataaaaaaatgttggaccttttaatcaaaatctgacctgatctggtccaattcggtccg
721 agtcaggtcaaggttgatcaattctgagaaagtgaaataagaaaaaaaattgtggtgttaa
781 aatttcatttcattgcatggcatctgtagagtgtttatatggtttatttccagttgcatg
841 ctgtaaattagtctctaattgcgttggttagttgggatgagttgttgatcaatgcaactt
901 attttttgcaggagtaggcaaaattcctactcaaattttcattaaactaaataaaaaag
961 tacaatagaaaaacccaaaaaaaaaaaaaaaattaaaagatgaagaagatgagaaggtct
1021 actcaaattttcatcaatgcagcttattttattgagaaggtctactttgttctaaaccct
1081 tgattttaaagaactaatagtgtcttagatgagttgtaagttgggtccattgattgtttc
1141 tcacctatttttctgtttagttggtggtagtttaagtttactatttgaaaatctgcacat
1201 tcctatggagggagaatctaaaaatgcttgtatctttcagtattaataatgttgtcgcta
1261 ctcggtctcttttccttccgaagatgccaaatacacatttgtcctttttttaatcacatt
1321 attggtgtgagcttttttttgacacttctgtaagaatccagagtaggaaagtgcacgtga
1381 acgttgtgcatgcgttaagagattacatgagataatttgtaacggtgtatgtgtaaagaa
1441 cgaatctatagaaatgcataaaggtgggccattttcaaaatattgaatcagctgatcata
1501 aacatcagttgcaccaaattttggtgtactgacataaattaccgttaactttgtaataag
1561 tttgtaccacccgtttatcttaagttcacccaattttccttgaataactcttatttacac
44 V D T R R K P F
1621 ccagcttgtccaatttctaacacactattttccagGGTTGATACCAGGAGAAAGCCGTTC
52 T P P R E V H V Q V T H S L S P Q K I E
1681 ACTCCGCCGCGTGAGGTGCATGTGCAAGTAACCCACTCCTTGTCCCCGCAGAAGATTGAG
72 M F K G L E S W A E D N I L P L L K P V

1741 ATGTTCAAGGGCCTTGAGTCCTGGGCTGAGGACAACATTTTACCCCTACTCAAGCCAGTG
92 E K C W Q P Q D F L P E P S S D E F Y D
1801 GAGAAGTGCTGGCAGCCTCAGGACTTCCTTCCAGAGCCTTCGTCCGATGAATTTTATGAC
112 E I N E I R L R A K E I P D D Y Y V C L
1861 GAAATTAACGAGATCCGATTGCGGGCAAAGGAAATCCCTGATGATTATTATGTCTGCTTG
132 V G D M I T E E A L P T Y Q T M L N T L
1921 GTTGGGGACATGATAACCGAGGAAGCTCTTCCGACATATCAGACTATGCTGAACACTCTG
152 D G V R D E T G A S L T S W A I W T R A
1981 GACGGGGTTAGGGATGAGACTGGAGCGAGCCTGACGAGCTGGGCCATCTGGACTCGTGCC
172 W T A E E N R H G D L L N K Y L Y L S G
2041 TGGACTGCTGAGGAAAATAGGCATGGTGATTTGTTGAACAAGTACCTGTACTTATCAGGT
192 R V D M K M I E K T I Q Y L I G S G M
2101 CGGGTTGACATGAAAATGATTGAGAAGACAATCCAATATCTTATCGGCTCTGGCATGgta
2161 agcaataatgtgattttggattttgtaactctgtaggatttcgctgtgttttactttgtt
2221 accaccaggtatactgattggcaaattgttctgcatgtgtgtgcatgcatgtctacagat
2281 tacgtgatcctgtttaggtaacagtggaaccagtttggtgagatgtgcgagtaagtacct
2341 gaaagagtgaagtaagaatagaatacagatccagtttgtttaagcacccaaaatcaagca
2401 ttcaaggaaagagataaatagataaattataaaatatagggttagatagttacctgaact
2461 ttgttttgacagctatagatgcagcactgctggcttttgtctttttaatgcgtacttaat
2521 attttcgctgagcaataaattattgtaattctactgttgaagcttacattaaatatttt
211 D P R T E N N P Y L G F I
2581 gtattttattttgactgacagGATCCAAGGACAGAGAACAACCCTTACCTGGGTTTCATT
224 Y T S F Q E R A T F I S H G N T A R H A
2641 TACACCTCATTCCAGGAAAGGGCAACATTCATTTCCCATGGTAACACTGCTCGTCATGCC
244 K D Y G D L K L A Q
2701 AAAGATTATGGGGACCTCAAGCTTGCTCAGgtaaagctctaacattatacctgattcatg
2761 cagtagtgatcttttcttttctctgatcattaatgtatttatttatattccaaataaatt
2821 aagtgtgtagcactgtattctctcttcttttatgttgctttctttctttctctttctatt
2881 tttttttttttgaggcggggtggggctaggccttacaaaaaatttagagtttgtaattcg
2941 ctcatgtttgattcgagaaaagttcgcaattagcttctgcgatataaattcgatctattt
3001 tcgaattaattcagttcgattcgaattggatacagatcaaaattgaagttacccctgttc
3061 aattcaataagttcgcaagccaaatatggctcaaattttaaagtcccccagttcaaccca
3121 atcagtagctttgatatgtccttctgatgaaagttaattttcattgaattgatattaata
3181 tctagttaagggtgaccgtcttcaactttctatattttgtttatccttcttatgatgggt
3241 gcacggttggtggcacacacatgtgacgctgaacatgctatgttttgatagctttttaat
3301 agtttcgagtttattatgaaccaaatcgaatcagctcgatttaaatcaaaccgaatacct
3361 tagacactcctcagatcgaaatcgaccatctatttcgaattggcttgattcgactaggta
3421 acaaactaaatatcctacagggctgattcaagtcagttcgatttgtgtgtgcccctaggt
3481 ggggcagagggttatttcacaggcttatgattaagttaatataattcacagttgttattg
3541 tgatgttatattccaatgatttaactgtatggaatataatgtgcgtctgtttgtactaaa
254 L C G M I A S D E K R H
3601 ttttaataatcaaaacttctgcagCTATGTGGGATGATCGCATCGGATGAGAAACGTCAT
266 E T A Y T K I V E K L F E L D P D G T I
3661 GAGACAGCCTACACAAAGATTGTTGAGAAGTTGTTCGAGCTCGACCCGGACGGCACCATT

286 L A L E D M M R K K I S M P A H L M Y D
3721 CTTGCTCTGGAGGACATGATGCGCAAGAAGATCTCTATGCCAGCACACCTGATGTATGAT
306 G V D D N L F E H F S N V A Q R L G V Y
3781 GGTGTTGATGACAACCTCTTCGAGCACTTCTCCAATGTGGCTCAGCGCCTCGGCGTCTAC
326 T A K D Y A D I L E F L V A R W K V A D
3841 ACTGCCAAGGACTATGCTGACATCCTTGAGTTCCTGGTGGCCCGCTGGAAGGTTGCTGAC
346 I T G L S G E G A K A Q E Y V C T L A P
3901 ATCACTGGTCTCTCTGGTGAAGGTGCAAAAGCGCAGGAATATGTGTGCACACTCGCCCCC
366 R I R R L D E R A Q S R A S K E R R T R
3961 AGGATCCGTAGGTTGGATGAGCGGGCTCAGAGCCGCGCCTCCAAGGAAAGGAGAACCAGG
386 P F S W I Y G R E V Q I *
4021 CCATTTAGCTGGATTTATGGAAGGGAGGTCCAAATT**TGA**

(B) *CeFAB2-2*

1 M A C R M A L N M D M H M L C S F T S P
1 **ATG**GCATGCAGGATGGCATTGAACATGGACATGCATATGCTCTGCTCCTTCACCTCCCCA
21 S K S K K G R R L G S I K V S M V D T S
61 TCAAAAAGCAAGAAAGGAAGGAGATTAGGTTCTATCAAGGTCTCCATGGTGGACACATCT
41 T T K
121 ACTACCAAgtaagctcatttccatttccattatgtataattgtatatatatgcatatgct
181 tcatatccttactataataaaagcataagggaaaaaatgacatgaattaaagggttatct
241 tccccaaataagaaacctgagccgttagtcattttcttgaacagatcagaaccgttgatt
301 tacgttaatgctcgttgacattaatgtctcaacccattaaacttaaggcccatatcaatt
361 tcctcaatataagacctattacatgttatcttaaaaatttattataagaaataacaattt
421 cagcccaaagcaacgcgtgagctcttttaattactagtacatataatatagttgctcgat
481 ttgtacgtcgatcatattcatcgcttcgatatcttgggtccgaggtttgcttcctgtagc
541 ccttcctgtgagctaaacagaatgtagttgctgaaatgttacttacagtgttatatagtt
601 aagaaattcacagaaaaatgcatccacaaaaacatataaagacagggcaattcatgcata
44 V E T A R K P F T P
661 aaatattgagtactgacacaatactttccagGGTCGAAACCGCAAGAAAGCCGTTCACTC
54 P R E V H A Q V T H S M P P E K I E M F
721 CGCCGCGCGAGGTGCACGCGCAAGTTACGCACTCCATGCCCCCAGAGAAGATTGAGATGT
74 K G L E S W A E D N I L P L L K P V E R
781 TCAAGGGCCTCGAGTCGTGGGCGGAGGACAACATTCTACCCCTACTCAAGCCGGTGGAGA
94 C W Q P Q D F L P E P S S D E F Y D E I
841 GGTGCTGGCAGCCTCAGGACTTCCTACCAGAGCCTTCCTCCGATGAATTCTATGACGAAA
114 K E I R L R A K E I P D D Y Y V C L V G
901 TAAAGGAGATCCGGTTGCGGGCGAAGGAAATCCCTGATGATTATTACGTCTGCTTGGTCG
134 D M I T E E A L P T Y Q T M L N T L D G
961 GGGACATGATAACCGAGGAAGCTCTTCCGACGTACCAGACTATGCTGAACACTCTGGACG
154 V R D E T G A S P T S W A I W N R A W T
1021 GGGTCAGGGATGAGACCGGAGCGAGCCCGACGAGCTGGGCCATCTGGAACCGTGCCTGGA
174 A E E N R H G D L L N K Y L Y L S G R V
1081 CCGCTGAGGAAAATAGGCACGGTGATTTGTTGAACAAGTACCTGTACTTATCAGGTCGGG
194 D M K M I E K T I Q Y L I G S G M

1141 TCGACATGAAAATGATCGAGAAGACGATCCAATATCTTATCGGCTCTGGCATGgtaatta
1201 ttaagcaataatgtgattttggattttgaacaataatgcatacttaatatatatttttgc
1261 gcgttgagcaataaatctataaattagaaagaagggtaagtctccaagcacctcaactat
1321 gccacatcatcaatgttattaaccattgattttgtttaaatctcttcatctactttgatc
1381 caatgatttatatttattctctctccccatttaataagatgatatgatatgtctaagaca
1441 aaatttaatccttaaagtggtccaacattatcatcatcatcatcatcattattcacatga
1501 aaagttttaaaattatatttaatttttttaaaataagtatttatattttataagatttatt
1561 atttttttgttatcatatttcgggggcctgagttacagctatagttactgtaattctac
1621 atttctttaccgttaaagcttacattaaatacatattttgtattttgttttggctgacat
211 D P R T E N N P Y L C F I Y T S F Q E
1681 gcagGATCCAAGGACAGAGAACAACCCCTACCTGTGTTTCATTTACACCTCATTCCAGGA
230 R A T F I S H G N T A R H A K D Y G D L
1741 AAGGGCAACCTTCATTTCCCACGGCAACACTGCTCGCCATGCCAAAGATTATGGGGACCT
250 K L A Q
1801 CAAGCTTGCTCAGgtaagtctttcctagctaacatcatacacatgccggagccagaaaat
1861 ttttatatggaattcaattattatactaaattttatgatatatataataatcacaattaa
1921 tataacaaataaaaaaattatggggttcactgtagaataatctttatatttttttatgggg
1981 ttcagaaacatattttcttaagaaattacttgtgtgacatgagaaattctatataaattt
2041 gtacaatttggatttttaatgggggacattgaaccccactgagtgaatgtatgtggatca
2101 tgcagtactcatctttccgatcgtttctttgatcatggatcgaatatatttctttctatt
2161 ccaaatgatttcagtgtgccttgttccaatcttcaacataaaacaagattaaattatata
2221 atgcacaattgttatatattgtgttatatatatatatatgttccaatgatccaacacaaa
254 L C G M I A A D E K R H
2281 atttaatggccataacttgtgcagTTATGCGGGATGATCGCGGCGGATGAGAAACGCCAC
266 E T A Y T K I V E K L F E L D P N D T I
2341 GAGACGGCCTACACGAAGATCGTCGAGAAGTTGTTCGAGCTCGACCCAAACGACACCATC
286 L A L E D M M R K K I A M P A H L M Y D
2401 CTTGCTCTGGAGGACATGATGCGCAAGAAGATCGCCATGCCAGCGCACCTGATGTACGAC
306 G V D D D L F E H F S N V A Q R L G V Y
2461 GGCGTTGATGACGACCTCTTCGAGCACTTCTCCAATGTGGCTCAGCGCCTCGGCGTCTAC
326 T A K D Y A D I L E F L V A R W K V A D
2521 ACTGCCAAGGACTACGCTGACATCCTCGAGTTCCTGGTGGCCCGCTGGAAGGTCGCTGAC
346 I T G L S G E G A K A Q E Y V C T L A P
2581 ATCACTGGCCTCTCTGGTGAAGGTGCAAAAGCGCAGGAATATGTCTGCACACTCGCCCCG
366 K I R R L D E R A Q S R A S N E R R R T
2641 AAGATCCGTAGGTTGGATGAGCGGGCTCAGAGCCGCGCCTCCAACGAACGGCGGCGAACC
386 R P F S W I Y G R E V H I *
2701 CGGCCGTTTAGCTGGATTTATGGAAGGGAGGTTCATATT**TGA**

(C) *CeFAB2-3*

1 M A C R M A L N M D M L C S F T S P I K
1 **ATG**GCATGCAGGATGGCATTGAACATGGACATGCTCTGCTCCTTCACCTCCCCAATAAAA
21 S K K G R R L G P L K V S M V D T T T T
61 AGCAAGAAAGGAAGGAGATTAGGTCCTCTCAAGGTCTCCATGGTGGACACAACTACTACC
41 K

121 AAgtaagctcatttccatttccattatgtataattgtatatatatgcatatgcttcatat
181 gtatatagttgctcgatttgtacgtcgatcatcgcttcgatatcttgggtccgaggtttg
241 gttcctgtagcctttcctgtgagctaaacagaatgcagttgctgaaatgttacttacagt
301 gttatatagttaagaaatttacagaaaaatgcatctacaaaaacatataaaaacagggca
361 attcataaaataataaactagctgatgacgagtatcagttgcattacattttgcaggatt
42 V E T T R K P Y T P P
421 aagtatatactgacacaatacttttcagGGTTGAAACCACAAGAAAGCCGTACACTCCGC
53 R E V H A Q V M H S M P P E K I E M F K
481 CGCGTGAGGTGCACGCACAAGTTATGCACTCCATGCCCCCAGAGAAGATTGAGATGTTCA
73 G L E S W A E D N I L P L L K P V E R C
541 AGGGCCTCGAGTCGTGGGCGGAGGACAACATTCTACCCCTACTCAAGCCGGTGGAGAGGT
93 W Q P Q D F L P E P S S D E F Y D E I K
601 GCTGGCAGCCTCAGGACTTCCTACCAGAGCCTTCCTCCGATGAATTCTATGACGAAATAA
113 E I R L R A K E I P D D Y Y V C L V G D
661 AGGAGATCCGGTTGCGGGCGAAGGAAATCCCTGATGATTATTATGTCTGCTTGGTCGGGG
133 M I T E E A L P T Y Q T M L N T L D G V
721 ACATGATAACTGAGGAAGCTCTTCCGACGTACCAGACTATGCTGAACACTCTGGACGGGG
153 R D E T G A S P T S W A I W N R A W T A
781 TCAGGGATGAGACCGGAGCGAGCCCGACGAGCTGGGCCATCTGGAACCGTGCCTGGACCG
173 E E N R H G D L L N K Y L Y L S G R V D
841 CTGAGGAAAATAGGCACGGTGATCTGTTGAACAAGTACCTGTACTTGTCAGGTCGGGTCG
193 M K M I E K T I Q Y L I G S G M
901 ACATGAAAATGATCGAGAAGACGATCCAATATCTTATCGGCTCTGGCATGgtaagcaata
961 gtgtgattttggagtttgcaactgtgtaggattgtgctgtgttttactttgttaccagta
1021 cctccaaagatactggtatactgattggtaaattatgctgcatgtgtgagcaataaatta
209 D
1081 ttgtgacattattgttgcagcttacattaaaataatttgtattttattttgactgacagG
210 P R T E N S P Y L G F I Y T S F Q E R A
1141 ATCCAAGGACAGAGAACAGCCCTTACCTAGGTTTCATTTACACCTCATTCCAGGAAAGGG
230 T F I S H G N T A R H A K D Y G D L K L
1201 CAACCTTCATTTCCCATGGTAACACTGCTCGTCATGCCAAAGATTATGGGGACCTCAAGC
250 A Q
1261 TTGCTCAGgtaagtccttcctaacatcatacatacctgattcattcatgcagtactcatc
1321 ttttcgtttctctgatcatggatatatttctttctattccaaataatttcagtgtgcctt
1381 gttccaattttcaacataaaacaagattaaattatataatgcacaattgttattgtgtta
1441 ttatattccaatgatccaacagtacaggattctgtgtccgtctgtttgtactaaaatcta
252 L C G M I A A D E K R H E T
1501 atggtcaaacttgtgtagTTATGCGGGATGATCGCAGCGGATGAGAAACGTCACGAGACA
266 A Y T K I V E K L F E L D P N G T I L A
1561 GCCTACACAAAGATTGTCGAGAAGTTGTTTGAGCTCGACCCAAATGGCACCATCCTTGCT
286 L E D M M R K K I S M P A H L M Y D G V
1621 CTGGAGGACATGATGCGCAAGAAGATCTCCATGCCAGCGCACCTGATGTACGATGGCGTT
306 D D N L F E H F S N V A Q R L G V Y T A
1681 GATGACAACCTCTTCGAGCACTTCTCCAATGTGGCTCAGCGTCTCGGCGTCTACACTGCC

326 K D Y A D I L E F L V A R W K V A D I T
1741 AAGGACTATGCTGACATCCTTGAGTTCCTGGTGGCCCGCTGGAAGGTTGCTGATATCACT
346 G L S G E G A K A Q E Y V C T L A P R I
1801 GGTCTCTCTGGTGAAGGTGCAAAAGCGCAGGAATATGTGTGCACACTCGCCCCCAGGATC
366 R K L D E R A Q S R A S K E R R T R P F
1861 CGTAAGTTGGATGAGCGGGCTCAGAGCCGCGCATCCAAGGAAAGGAGAACCAGGCCATTT
386 S W I Y G R E V Q I *
1921 AGCTGGATTTACGGAAGGGAGGTTCAAATT**TGA**

(D) *CeFAB2-4*

1 M A C I M A L N I N M L C S S P P W K S
1 **ATG**GCATGCATCATGGCATTGAACATTAACATGCTCTGCTCCTCCCCTCCATGGAAAAGC
21 N K G R K F G P L R V S V G D T I T K
61 AACAAAGGAAGGAAGTTTGGTCCTCTCAGAGTCTCTGTGGGGGATACAATTACAAAgtaa
121 gctcattaatttaattacttttcgattatgtttataatcaagaacttaatgaccatttga
181 atggtctaatatctgtttttcatttttttttgtatctttttttttatgcatatcagtaatac
241 taataatagaaaggtttaaaagaagaaatctttagtttaattacatgttaaacaatgtat
301 tttagtccatgttttcgcaaagtacctgttctagttttgtcaatttgaacaacgtttcgg
40 A H T V K N P V G
361 gacgttaacatgttgatttgactttccaatgaagGGCTCATACAGTGAAGAATCCAGTCG
49 S P C K S K D R R I T H S M P E E K I E
421 GCTCGCCGTGCAAGTCAAAAGATCGGCGAATAACTCATTCCATGCCAGAAGAGAAGATCG
69 I I K N L E P W V R D N I L P L L K P V
481 AGATTATCAAGAACCTCGAACCGTGGGTTCGGGACAACATTTTGCCGCTCCTGAAACCCG
89 E K C W Q P Q E F L P K P S T E G F Y E
541 TCGAGAAGTGCTGGCAGCCGCAAGAATTTCTTCCGAAGCCCTCCACTGAGGGCTTCTATG
109 Q V R E I Q L R A K E I P D D C F V C L
601 AACAAGTGCGAGAGATCCAATTGCGAGCAAAGGAAATTCCGGATGATTGTTTTGTATGCC
129 V G D M I T E E A L P T Y Q T W I N G L
661 TGGTAGGGGACATGATAACGGAAGAGGCGTTGCCTACGTACCAAACTTGGATAAACGGCC
149 D G V G D E T G V D P S S W G I W T R G
721 TTGACGGTGTCGGAGATGAGACCGGGGTCGACCCCTCGAGCTGGGGAATATGGACCCGGG
169 W T A E E N R H G D L L N K Y L Y L S G
781 GATGGACTGCGGAGGAAAACCGGCATGGCGATTTGTTGAACAAGTATCTGTACTTGTCAG
189 R V D M K M V E R T I Q Y L I G S G M
841 GCCGTGTCGACATGAAGATGGTCGAGAGGACGATACAGTATCTCATCGGCTCTGGGATGg
901 taagttctttatttgtgtttttttcgtacgtctttcagtgattgagtatgctgaattctc
961 agtttgtgacgtcttattgtcaatttgttaccattgccgggtttccagttcaaatgcata
1021 tgtaataggtgatcctttttaccagtttactggactgaggaaacttcaatgtggctaaaa
1081 gttacttgcttgtatccaaatattttctatagtttttaatttcgacgtacacgatttgct
1141 gatcaaaataacgggaacgcacatatataatcaagcttatcgaagaaatccagtatcaga
1201 actccaaacaagttaaagactgacgacaaacagatcgaactgatgtaaaaggcgaaagtt
1261 aaaccggtaaggcggcaaagaagtttccttaatcaagattgcataattaatcaactgatc
1321 aacagctcaaggaattgaaaagttaaaagatcctgtctttttttcaaagacaggaaaaca
208 Y L R T E N N P Y M

1381 atactgaaactttttttgttatgatttgcagTATCTGAGAACAGAGAACAACCCATACATG
218 G F I Y T S F Q E R A T F I S H G N T G
1441 GGCTTCATATACACCTCATTTCAGGAGAGAGCAACCTTCATTTCCCATGGAAACACCGGC
238 R L A K E H G D V R L A Q
1501 CGTCTTGCCAAAGAGCACGGCGACGTCAGGCTCGCGCAGgtattacattgcctgtgaact
1561 tgtttcttttttttccttcaattttggcctgtgatcattttaaaatagttttaattcacta
1621 ttattattagtttaatcttgcttgctttaattaatacattgttacagtttgtaatatgta
1681 cagttaataactgttgattctggttcaccttgactctcgctttgaaccgatctcgaagta
1741 cttcagtgctttactgatctagcatatatagttcacattcttaattccgaggatcgatcc
1801 tgccctcgtgttatactttgttcaattttgcacaacagattatgaagaactaacaaacac
1861 aagaaataaaagtataaatctcgatgttttcgatctgtgagagggagaatatatctctga
1921 aagttattctaattcaacagtagacacttaaaactaaattctctctcaaaagatcagaac
1981 tttcttgtttctcctcctccgatcttcagttttcgaggtgctctcgagatgctgtctcaa
2041 acatacacacattcttgctcatttcatctttttcgagctcggcttccatttgattcgaac
2101 tcgaaattccattgtgttgctgttgctgtgttcttttgagtttcacttcgagatcaaaca
2161 aacaactacacattattttgtcaaacgcataatccatatcgattgaatctttaaaattat
251 I C G T I A S D E K R H E N A Y S K I
2221 ttcagATATGTGGAACAATTGCATCCGATGAGAAGCGTCACGAGAATGCATACAGCAAGA
270 V E K L F E V D P D Y T V L A L A D M M
2281 TCGTGGAGAAGTTATTCGAAGTCGACCCCGACTACACCGTGCTTGCTCTGGCAGACATGA
290 R K K I V M P A H N M Y D G T N D N L F
2341 TGCGTAAGAAGATCGTGATGCCTGCGCACAATATGTACGATGGTACCAACGACAACCTCT
310 A H F S L V A Q R I G V Y T S K D Y A D
2401 TCGCTCACTTTTCCTTGGTGGCACAACGCATCGGCGTCTATACATCCAAGGATTATGCTG
330 I L E F L L A R W K V A E L A G L S S E
2461 ACATCCTCGAGTTCCTGCTGGCACGCTGGAAGGTCGCTGAACTCGCAGGTCTTTCAAGCG
350 G R R A Q E Y L C T L A P R F R R L E E
2521 AAGGTAGACGGGCACAGGAATACCTGTGCACTCTCGCTCCCAGGTTTCGAAGGCTGGAGG
370 R E Q R R R A G D G E T V S F S W I Y G
2581 AGCGTGAACAGAGACGTCGTGCCGGGGATGGAGAGACCGTTTCGTTTAGCTGGATTTATG
390 R G V Q I *
2641 GCAGGGGGGTTCAAATC**TAA**

(E) *CeFAB2-5*

1 M A F Q V A S E M V F I S S C S P Q F G
1 **ATG**GCTTTCCAAGTAGCTTCAGAAATGGTGTTCATTTCCTCCTGCTCTCCCCAATTTGGA
21 S Y K R R H M S S I K V Y M S D T K T K
61 AGCTACAAAAGAAGACACATGAGCAGTATCAAGGTTTACATGTCAGATACAAAAACAAAg
121 tgagttctttttacttttcagtcatataaaaattttcagttttctttcactatgattatc
181 atataaatacaattgactacatcactttgttctgttgtctttcctgttgcatgcattgtt
241 ctctaagcatttctgtcctgcagaggacaatgtattattcactcagttgccttggctctg
301 atacttgatgttttgtctttgtgctgatacttgctataatttgttatgtcaacctttttgg
361 tttttgcacaaattgtactttcttctagaattttccatcaactaacttggaagagttcg
421 tatttcttgatttctctgatattaatttaaccttgcatgcattcactttttttcctttgtg
481 ggggcagtgtgtgtgttcagatatatgtgcatagcataacaccagtgtcatgaaatttcc

541 tatttgagagaatatatgtatgccaagttctgcccattaggccattacctccctattgga
601 aagtgaacacagagaagtctgggcaacttcagtcatatcatttttttttttcatataggtg
661 cacatgcaaggaaagaagaaatacatctataattaatcacatttcaaagagatgaaaact
721 gtaaattgtatatggaatggttacaattttgagacagtttgaggccaactgtgtatgatt
781 tggatcgattctaactgaagttggtccaactgtgaactgtcgtaattagttaacatttat
841 tattatagatacattaattcagacttcaattaaactctgaaaagacagataagaacagta
901 acataagaaaatggaagagaaacttaaataaactttaattttaattaattgccactggtt
41 P S C
961 ctaatacaagtaattgtttttttgccttttttatgggttcaaactgagaacagGCCGTCCTG
44 A P N N L R T Q I T H A M P Q E K I E I
1021 TGCGCCAAACAACTTGCGCACGCAAATAACTCATGCTATGCCTCAAGAGAAGATCGAGAT
64 F N E L E P W A R D N I L P L L K P V E
1081 ATTTAACGAGCTCGAGCCGTGGGCCAGAGATAACATCTTGCCGCTGCTCAAGCCGGTAGA
84 K C W Q P Q D F L P D A S S T D E G F Y
1141 GAAATGCTGGCAGCCACAAGACTTCCTTCCGGATGCTTCGTCTACCGATGAGGGGTTTTA
104 D Q V R E I Q L Q A K E I P D D Y F V C
1201 CGATCAAGTGCGGGAGATTCAGTTGCAGGCAAAGGAAATCCCGGACGATTATTTTGTGTG
124 L V G N M I T E E A L P T Y Q T M L N T
1261 CTTGGTTGGTAACATGATAACCGAGGAAGCACTGCCGACATACCAGACAATGCTGAACAC
144 M E G I R D E T G V S P M S W A I W S R
1321 CATGGAGGGGATTAGGGATGAAACTGGTGTGAGCCCGATGAGCTGGGCAATTTGGTCCCG
164 C W T A E E N R H G D L L N K Y L Y L T
1381 TTGCTGGACTGCTGAGGAGAACCGGCATGGCGATCTGTTGAACAAGTACCTGTACTTAAC
184 G R V D M K M V E K T I Q Y L I S N G M
1441 GGGTCGTGTGGACATGAAGATGGTTGAGAAGACGATACAATATCTCATCAGTAACGGAAT
204
1501 Ggtatggaatgatatgatttttttgtattatcactatatattatgtacatttttagtgtct
1561 tatattactatggatcgtgttcataccggcattgttactgacacgctttgggtacctaac
204 D
1621 tttcgaaagtatgctagcttcaagcattcatgtatgaacaacattcttatgtttcagGAT
205 V G L E Q N P Y H G Y I Y T S F Q E R A
1681 GTGGGTCTTGAACAGAATCCGTACCACGGTTATATATATACCTCGTTCCAAGAAAGAGCC
225 T F I S H S N T A R H A K K Y G D L K L
1741 ACTTTCATTTCGCATAGCAACACTGCTCGCCATGCCAAGAAGTATGGAGATCTTAAGCTT
245 A Q
1801 GCTCAGgtactactttgcatatgtattatgcaaaactttttaattttcttattattatag
1861 tgtcatattttaaagtctgaaaaaataatagtaaggaacctccaattaataaaagcaatg
247 I C
1921 taaattaggacagtttaatttatattaaattacgattaacaaaacttttcctcagATATG
249 G I I A S D E K R H E T A Y T K I V G K
1981 TGGGATAATCGCATCAGACGAGAAGCGTCATGAGACAGCTTACACAAAGATTGTGGGTAA
269 L F E V D P D S T V L A L A N M M Q H K
2041 GCTGTTTGAGGTTGACCCCGACAGCACAGTTCTGGCACTGGCAAACATGATGCAACACAA
289 V T M P A H L M Y D G C D T G L F K N Y

2101 AGTCACAATGCCAGCACACCTGATGTACGACGGCTGCGACACTGGCCTCTTCAAGAACTA
309 S M V A Q R L Q V Y T A K D Y A D I L E
2161 CTCAATGGTGGCGCAGCGCCTCCAAGTTTACACTGCCAAGGACTATGCTGATATCCTTGA
329 F L V Q R W K V S D L V G L S G E G R R
2221 GTTCCTGGTGCAGCGCTGGAAGGTGAGCGACCTTGTTGGTCTCTCTGGTGAAGGAAGAAG
349 A Q D Y V C N L A P R I R K L E E R A Q
2281 GGCACAGGATTATGTGTGCAATCTTGCTCCAAGGATTCGGAAGCTTGAGGAAAGGGCTCA
369 N R A S K E Q K T R P F S W I Y G R E V
2341 GAACCGCGCCAGCAAGGAGCAGAAGACCCGGCCGTTTAGTTGGATTTATGGAAGAGAAGT
389 I L *
2401 CATACTG**TGA**

(F) *CeAAD1*

1 M Q A G N T F I T R H V A S P A V R M A
1 **ATG**CAGGCTGGCAACACCTTCATTACACGCCATGTGGCGAGCCCGGCGGTCAGGATGGCT
21 P Q K R Q C R V A A V L T A P P L R H N
61 CCGCAGAAGAGGCAATGCCGGGTGGCAGCAGTGCTAACAGCGCCACCACTTCGCCATAAC
41 V T H S L P P E K A E V F K S L E G W A
121 GTGACACATTCGCTTCCACCGGAGAAGGCTGAGGTGTTCAAGTCACTGGAAGGATGGGCC
61 K E S L L P L L K P V E D C W Q P T D F
181 AAGGAATCGCTGCTGCCTCTTTTGAAGCCGGTCGAGGATTGCTGGCAGCCGACAGATTTC
81 L P D S S K S T D E F Q E E
241 TTGCCCGATTCGAGCAAATCCACTGATGAATTCCAGGAGGAGgtaatgtatcatgtctga
301 atgatgattaattatctatgcaatgtatgttgtgtgacaccatgatcatgatgaattcaa
361 attttgaaattcaagcagagaaatgttgtctaaattttagattaattttggaaaaatagt
95 I R A L R
421 atctgatccagacttctccggcacggtaatgcgaataatttatagATTCGAGCGTTGCGG
100 E R T N E L P D E Y F V V L V G D M I T
481 GAAAGGACTAATGAATTGCCAGACGAGTACTTTGTGGTGCTTGTGGGGGACATGATAACC
120 E E A L P T Y Q T M I N T L D G V K D E
541 GAGGAGGCATTGCCGACATATCAAACCATGATCAACACACTCGATGGGGTTAAGGACGAG
140 T G A S S S P W A V W T R T W T A E E N
601 ACCGGGGCCAGCTCGAGCCCATGGGCGGTGTGGACCCGTACATGGACCGCCGAAGAAAAC
160 R H G D L L G K Y L Y L S G R V D M R M
661 CGGCATGGAGATTTGCTCGGGAAGTACCTGTACTTGTCGGGTCGGGTTGACATGCGTATG
180 V E K T V Q Y L I G S G M
721 GTAGAAAAGACAGTTCAGTACCTAATTGGATCAGGAATGgtatgtcgaatactgatagtg
781 tgtaccagtatcttgtatttaattaatactgagcttgtttattcagaaacttcatgcgtc
841 cattgatggacagtccatagaagttacactgtgagtgtaactgttacatctgggcccata
901 tagggtctacttgtcatactgtttgttacttctatggactatccatcgatgaacggtagg
961 tcttactgattgtttattttgatgtttgacattaatttcagtttttatatgttgattaaa
193 D P G T E N N P Y L G F V
1021 tattttgatttctgttaccagGATCCAGGCACTGAGAACAACCCATATCTGGGTTTCGTG
206 Y T S F Q E R A T A V S H G N T A R M A
1081 TACACGTCGTTTCAGGAGCGAGCTACTGCAGTTTCTCACGGCAACACTGCCAGGATGGCA

226 V G A G D I T L A R A C G T I A A D E K
1141 GTCGGAGCTGGCGACATAACTCTTGCCCGTGCTTGTGGTACAATCGCCGCAGACGAGAAG
246 R H E R A Y Q A I I E Q L L Y L D T D T
1201 CGGCATGAGCGTGCGTACCAAGCCATCATCGAACAATTGTTGTACCTTGATACCGACACC
266 A M L A I A D M M H K K I T M P A H L M
1261 GCCATGCTCGCCATTGCTGACATGATGCACAAGAAGATCACCATGCCCGCACATCTCATG
286 Q
1321 CAGgtacgtacgtaccgactgatttttaaaaacttaatatcaaatcatatatcactttta
1381 gagtggttatgctatggatcggctaagcaacatttcattttgaatcgaacagttattatc
1441 aagcacatctcattaattgtatctaaaattcatatcgtgaccatctgattatgaaaaatg
1501 aatatttaagtagtctggtacattgtctatgttctaaattctaaactaagatataaacac
1561 ataattcaaaacaatgcttacacagtcacactgtataataactgtttatttggataattc
287 D G K D P N L F E H Y S S V A Q R L G
1621 atagGATGGAAAAGACCCAAATCTGTTTGAGCACTACTCATCAGTGGCGCAACGGCTAGG
306 V Y T V K D Y A D I V E H L V N R W R L
1681 TGTGTACACTGTCAAAGACTATGCAGACATTGTGGAGCATCTGGTTAACAGATGGAGGCT
326 E Y L Q D G L S S E G R R A R D F V C G
1741 GGAGTACCTGCAAGATGGGCTGAGCAGTGAGGGCAGGCGAGCCCGGGACTTCGTCTGCGG
346 L P Q R M R R M Q E K F E D R A R K N R
1801 GCTCCCTCAGAGGATGAGGAGGATGCAGGAGAAGTTCGAGGATCGGGCCCGGAAGAACCG
366 G V S N G P D T V P F S W V F G R Q V K
1861 GGGGGTTTCGAACGGGCCCGACACCGTTCCTTTTAGCTGGGTCTTTGGTAGGCAGGTTAA
386 V *
1921 GGTG**TAG**

(G) *AtFAB2*

1 M A L K F N P L V A S Q P Y K F P S S T
1 ATGGCTCTAAAGTTTAACCCTTTGGTGGCATCTCAGCCTTACAAATTCCCTTCCTCGACT
21 R P P T P S F R S P K F L C L A S S S P
61 CGTCCGCCAACTCCTTCTTTCAGATCTCCCAAGTTCCTCTGCCTCGCTTCTTCTTCTCCG
41 A L S S G P K
121 GCTCTCAGCTCCGGCCCCAAgtcagtctctttctctccatcatcgtctctcttatctact
181 ttcgattctctctgtttcatctttgtttactgtgttctgggttttgcttgttcttatcga
241 tattggatctacgttttctcgattttgttcagtgttatttgcttacatttgcttaatctc
301 gagtgtgatttcaataaaagtacgttcttttaatctgggtttataagaatgggccatcct
361 ctgttctcaggtgattcattaatgctctttttcattgaatctgatgtttgttgattgttc
421 cctattattctcacgtttctctgagattgccccatctatcaaatcgttgcctttaccata
481 tttattctgcatcagattatgtagattgctcgttgattggttgttagttaagattaacac
541 tagttatcttctttatggtataatgatctttagattggttaagacttaacagacattgag
601 gaaagcttcttagtcaaagagaaagttttgttttgttttctctggtttagtaattttaa
661 tggtgaaatgtttatccacttgtttcttactcatttgatgagttttctcttttgtattga
721 caagagattcttgaacgtatcagtttacgtctgtttatctttcttcaaagacattacgtt
781 ggatatcacgcttcgtggcctttctctatgcatgcgtacttcgtggcttctctctttagt
841 ttgcgtccaaaattttcacacttttctgtaatttattattcattctttctctgttttgaa

```
 901 gacatttggctctttatcttgtctttgtctgatcagtagtcactacatttgtttctttca
 961 caatctttctttttttgcttcatattgaactgcaagaccaatccatgaatgcaaatataaa
1021 atattgtcttacggttaaccatctctaagtgtttgctagtcatgaattgtgaaattgttt
1081 cgcttcaaacatagaattagttgatttggaaatgtagacatgaatgcttatctaaataca
1141 ataaactgtgttcttgtttcagttgtgtatcagagatgagaccttgactggtaatttcaa
  48                                                     E  V  E  S
1201 tggagcataattaaatgctaatgatacttattttctggttaatgcagGGAGGTTGAGAGT
  52 L  K  K  P  F  T  P  P  R  E  V  H  V  Q  V  L  H  S  M  P
1261 TTGAAGAAACCATTTACGCCACCCAGGGAAGTGCATGTTCAAGTCTTGCACTCCATGCCA
  72 P  Q  K  I  E  I  F  K  S  M  E  N  W  A  E  E  N  L  L  I
1321 CCTCAAAAGATCGAGATCTTCAAATCTATGGAAAACTGGGCCGAGGAGAACCTTCTGATT
  92 H  L  K  D  V  E  K  S  W  Q  P  Q  D  F  L  P  D  P  A  S
1381 CACCTCAAGGATGTGGAGAAGTCTTGGCAACCCCAGGATTTCTTGCCTGACCCTGCATCA
 112 D  G  F  E  D  Q  V  R  E  L  R  E  R  A  R  E  L  P  D  D
1441 GATGGGTTTGAAGATCAGGTAAGAGAGTTAAGAGAGAGGGCTAGAGAGCTCCCTGATGAT
 132 Y  F  V  V  L  V  G  D  M  I  T  E  E  A  L  P  T  Y  Q  T
1501 TACTTTGTTGTTTTGGTGGGGGACATGATCACAGAAGAAGCACTTCCGACCTATCAAACT
 152 M  L  N  T  L  D  G  V  R  D  E  T  G  A  S  P  T  S  W  A
1561 ATGTTGAACACTTTGGATGGAGTTAGGGATGAAACAGGTGCTAGTCCTACTTCATGGGCT
 172 I  W  T  R  A  W  T  A  E  E  N  R  H  G  D  L  L  N  K  Y
1621 ATTTGGACCAGAGCTTGGACTGCAGAAGAAAACCGACATGGCGATCTTCTGAATAAATAC
 192 L  Y  L  S  G  R  V  D  M  R  Q  I  E  K  T  I  Q  Y  L  I
1681 CTTTACTTGTCTGGTCGTGTTGACATGAGGCAGATCGAAAAGACCATTCAGTACTTGATT
 212 G  S  G  M
1741 GGATCTGGAATGgtgagatagtttcaggcaattatcatgatttcttggttaatataacta
 216                            D  P  R  T  E  N  N  P  Y  L  G
1801 cctaatcgctttaacttatctttcatagGATCCGCGGACAGAGAATAACCCCTACCTTGG
 227  F  I  Y  T  S  F  Q  E  R  A  T  F  I  S  H  G  N  T  A  R
1861 CTTCATCTATACGTCATTCCAAGAAAGAGCGACATTCATCTCTCACGGAAACACAGCCCG
 247  Q  A  K  E  H  G  D  I  K  L  A  Q  I  C  G  T  I  A  A  D
1921 CCAAGCCAAAGAGCACGGGGACATCAAACTAGCCCAAATATGTGGCACAATAGCTGCAGA
 267  E  K  R  H  E  T  A  Y  T  K  I  V  E  K  L  F  E  I  D  P
1981 CGAGAAGCGTCATGAAACAGCATACACCAAGATAGTTGAAAAGCTCTTTGAGATTGATCC
 287  D  G  T  V  M  A  F  A  D  M  M  R  K  K  I  S  M  P  A  H
2041 TGATGGTACTGTCATGGCTTTTGCAGACATGATGAGAAAGAAAATCTCAATGCCTGCTCA
 307  L  M  Y  D  G  R  N  D  N  L  F  D  N  F  S  S  V  A  Q  R
2101 CTTGATGTATGATGGGCGCAACGACAACCTCTTTGACAACTTCTCTTCCGTGGCTCAGAG
 327  L  G  V  Y  T  A  K  D  Y  A  D  I  L  E  F  L  V  G  R  W
2161 GCTCGGTGTTTACACCGCCAAAGACTATGCAGACATTCTTGAGTTTCTGGTTGGTAGGTG
 347  K  I  Q  D  L  T  G  L  S  G  E  G  N  K  A  Q  D  Y  L  C
2221 GAAAATCCAGGACTTAACCGGGCTTTCAGGTGAAGGAAACAAAGCACAAGACTATTTATG
 367  G  L  A  P  R  I  K  R  L  D  E  R  A  Q  A  R  A  K  K  G
2281 CGGGTTGGCTCCAAGGATCAAGAGATTGGATGAGAGAGCTCAAGCAAGAGCCAAGAAAGG
 387  P  K  I  P  F  S  W  I  H  D  R  E  V  Q  L  *
```

2341 ACCCAAGATTCCTTTCAGTTGGATACACGACAGAGAAGTGCAGCTC**TAA**

(H) *AtAAD1*

1 M V M A M D R I A L F S S S S S V Y H H
1 ATGGTTATGGCTATGGATCGGATCGCTTTGTTCTCTTCCTCATCATCTGTTTATCATCAC
21 G S S H S H G S K S S R V F T I R S D S
61 GGTTCTTCTCATTCTCATGGCTCCAAATCTTCCAGAGTTTTCACTATTCGTTCCGACTCT
41 T
121 ACgtgagtttctctcttcatttccagaaattctgtttatttttctgtttccccaaaattt
181 tcctgattttgaggtttcgttctttctggggtttaagtttgttcagcgatgatcattatt
241 aacattggctttttatatgcaatctcctgcttctatcaaatttgtatttaaatgcagatt
301 tatttcgttctgtgattactaatctaaagcattggcttttgatcatacgcttgtgtgtgc
361 tctgttcttacgtattgcttaaaatgatcattatatttatagttagttacttttatttaa
421 agcattatagttacttttatcatatatatcttctcagtttgttttctgctgaagctatgg
481 acatgtcacatgtttgcgatcacatgtttgacatcgcaaaactcttaatttttgcattta
541 aaaaaaaaatctgatagggaaattgcaatctttgtctttcttgtacaattcagtatttgt
601 aatcactagtttatggagtatatgcatgcatggattgttccctttttttgactaaactat
661 tatttaatttggttgttggcctatcaggctacaatgttccatcaatgctttcttaattga
721 ttgctagtgtaagtctttttaaaatcaaatcacattccacataaattggttaaggctttt
781 taatattgtcttgactttttgtagattgcgtttactctatgtttatgcatatgcacacat
841 ctatgtatgtgtttatatgcactgtagttggtcaagttaggtctgaaatgtttttgcttg
901 ttgttttggtgatactgttttggtcagaagaaattttgatttagagcatgctttgtcttg
961 tagtactgcaagtgccttcactgttgttatatcatctatgatgaacactgaatcacacat
1021 tagcttgtctttttcttttaagaaactgattattctcgtgttgggatatctcctttttgg
1081 aggtgtcttaaagactataacttgtttgcagttggttacctgaataacacgaaaccctat
1141 catctaattctggtggctgtgttaaatgttaattataacagtattttcagtctctcaagt
42 A V G
1201 ttcttattctattgatcattttggaatcttctttaatggaaaaattgcagAGCGGTTGGA
45 R K L Y I P P R E V H L Q V K Y S M P P
1261 AGGAAACTGTATATTCCTCCTCGAGAGGTTCATCTTCAAGTGAAGTATTCAATGCCACCA
65 Q K L E I F K S L E G W A N D N L L A Y
1321 CAAAAGCTGGAGATTTTCAAGTCCTTAGAAGGATGGGCTAATGACAACTTGTTGGCTTAC
85 L K P V E K S W Q P T D F L P E P E S E
1381 TTAAAACCTGTTGAGAAATCATGGCAACCAACTGATTTTCTCCCTGAACCTGAGTCAGAA
105 G F Y D Q V K E L R E R C K E L S D D Y
1441 GGATTCTATGACCAAGTTAAGGAGTTAAGAGAAAGGTGTAAGGAGCTCTCTGATGACTAC
125 L I V L V G D M I T E E A L P T Y Q T M
1501 CTTATAGTGCTTGTTGGTGATATGATCACAGAGGAAGCACTTCCGACTTATCAGACCATG
145 I N T L D G V R D E T G A S P T P W A V
1561 ATTAATACATTGGACGGGGTTAGAGATGAGACGGGAGCAAGTCCAACTCCTTGGGCAGTA
165 W T R A W T A E E N R H G D L L N K Y L
1621 TGGACTAGGGCATGGACTGCGGAAGAGAATAGACATGGTGATCTTCTTAACAAGTACCTT
185 Y L S G R V D M R Q I E K T I Q Y L I G
1681 TATCTGTCTGGACGAGTAGACATGAGACAGATTGAAAAAACTATTCAATACCTCATTGGT

205 S G M
1741 TCTGGAATGgtaagctcctttagtataataatatggctagcttttcttataaaggctaaa
208 D P K T
1801 cctacaagtctctttttttggtcgtattgattgtgttttgttttgtagGATCCAAAAACT
212 E N N P Y L G F I Y T S F Q E R A T F I
1861 GAAAACAATCCTTACTTGGGATTCATATATACTTCTTTTCAAGAAAGAGCTACATTCATC
232 S H G N T A R L A K D L G D L T L G K I
1921 TCCCATGGAAACACTGCTAGACTGGCAAAGGATCTTGGAGACTTGACACTTGGGAAGATT
252 C G T I A A D E R R H E H A Y T K I V E
1981 TGTGGCACCATTGCTGCTGATGAGAGGCGCCATGAACACGCCTACACCAAGATTGTAGAG
272 K L F E I D P D T T V V G F A D M M R K
2041 AAGCTCTTTGAAATTGATCCTGATACCACAGTCGTGGGCTTTGCTGACATGATGAGGAAA
292 K I S M P A H L M Y D G R D D N L F D H
2101 AAGATCTCGATGCCTGCTCATTTGATGTATGATGGTCGTGATGATAATCTATTTGATCAC
312 F S S V A Q R L G V Y T A K D Y A D I L
2161 TTCTCGTCCGTGGCCCAGAGGCTTGGTGTCTACACTGCCAAGGACTATGCTGATATACTG
332 Q H L V E R W N V E K L S D L S S E G N
2221 CAACATCTTGTGGAAAGGTGGAATGTGGAGAAGTTGTCAGACCTTTCTAGTGAAGGAAAC
352 R A Q
2281 AGGGCCCAGgtaacaacattagaaccatgaaaccgtttttgttttcttgtttttgacttg
2341 acatgaaacatccaacttctattaagtttgtttactttgttaaaaaacctcaccttgcat
2401 taaggaacttacagaacctatcatatctataattgcattgtaacttctcctgtgcacttg
2461 gagattcatcaaattggaatctgagttctttacactctactctgacttttcgctttgttt
355 D Y L C G L P A R I R K L E E
2521 taatggcttcgaacagGACTACCTCTGTGGATTACCTGCTAGAATCCGCAAACTTGAAGA
370 R A Q G R T K E A A K N I P F S W I F G
2581 GAGGGCTCAAGGGAGAACCAAAGAAGCAGCAAAAAACATACCATTCAGCTGGATTTTTGG
390 R E V R A *
2641 TCGAGAAGTCAGGGCT**TAA**

(I) *AtAAD2*

1 M K M A L L L N S T I T V A M K Q N P L
1 **ATG**AAGATGGCTCTTCTCTTGAACTCGACGATAACGGTGGCTATGAAGCAGAATCCGCTG
21 V A V S F P R T T C L G S S F S P P R L
61 GTCGCGGTATCTTTTCCGCGGACTACTTGCTTGGGATCTAGTTTTTCTCCTCCTCGTCTT
41 L R V S C V A T N P S
121 CTTCGAGTTTCGTGTGTTGCCACAAACCCTAGgtgagttttatctttttagatcttcaat
181 tcgtttccttgatcaagtttttctttttctttttcttttttcatttggctttatact
241 ggttaatttcgttgtatatgtttaactcattggttatatttaactatttgtctaattggc
301 aaatcctttcaaaaccaaatcgaaacggtacaaaactgatcaaaaagttagtaattttttt
361 tccatatagaaaaatggcactgaatagtgaaagtgaacaacattgaacttagaaatactt
421 caaattaatctatagaattgattgtgttataagaaaatcctacaatcatttgcactcaaa
481 cggaaaaatcgcatgaagtcaggtaaactcattgggttttaaatacttgcatattaactt
541 atgttatactattacaataagacacgaacctgatatgaaaataacaattttcttacaatg

52 K T S E E T D K K K F R P I K E

601 aaaatgttatagCAAGACGAGTGAAGAAACAGACAAGAAAAAGTTTCGACCCATCAAAGA

68 V P N Q V T H T I T Q E K L E I F K S M

661 AGTACCAAACCAAGTGACCCACACAATAACACAAGAGAAACTGGAGATCTTCAAATCAAT

88 E N W A Q E N L L S Y L K P V E A S W Q

721 GGAGAATTGGGCCCAAGAAAACTTATTATCTTACCTCAAACCCGTCGAAGCTTCCTGGCA

108 P Q D F L P E T N D E D R F Y E Q V K E

781 ACCACAAGACTTTTTGCCGGAAACCAACGACGAAGACCGATTCTACGAACAAGTAAAAGA

128 L R D R T K E I P D D Y F V V L V G D M

841 GCTAAGAGATCGAACAAAAGAGATCCCTGACGATTACTTTGTAGTTCTTGTCGGAGATAT

148 I T E E A L P T Y Q T T L N T L D G V K

901 GATCACCGAAGAAGCTTTACCAACTTATCAAACTACTTTGAACACTCTCGACGGCGTAAA

168 D E T G G S L T P W A V W V R A W T A E

961 AGACGAGACCGGTGGTAGTCTAACGCCGTGGGCGGTTTGGGTTCGAGCATGGACGGCGGA

188 E N R H G D L L N K Y L Y L S G R V D M

1021 GGAAAATCGACACGGTGATTTGTTGAATAAGTATCTTTACTTATCAGGTCGTGTTGACAT

208 R H V E K T I Q Y L I G S G M

1081 GCGACATGTTGAAAAGACTATTCAATATCTTATCGGATCCGGCATGgtacgttgcactcc

1141 cataaatatatgtacgcttaaaatgtaaattataatttcttgaggatcaaattgataatg

223 D S K F E N N P Y N G F I Y

1201 tttataattatttttcagGACTCCAAATTTGAGAACAATCCCTATAATGGCTTCATCTAT

237 T S F Q E R A T F I S H G N T A K L A T

1261 ACTTCTTTTCAAGAGAGAGCCACTTTCATCTCCCATGGCAACACGGCGAAGCTAGCCACA

257 T Y G D T T L A K I C G T I A A D E K R

1321 ACCTACGGCGACACAACTCTCGCCAAAATCTGCGGAACAATCGCGGCGGATGAGAAGCGG

277 H E T A Y T R I V E K L F E I D P D G T

1381 CATGAGACGGCGTATACACGGATCGTCGAGAAGCTTTTCGAGATCGATCCCGATGGTACC

297 V Q A L A S M M R K R I T M P A H L M H

1441 GTACAAGCTCTAGCGAGTATGATGAGGAAGCGAATCACGATGCCGGCTCATCTGATGCAC

317 D G R D D D L F D H Y A A V A Q R I G V

1501 GACGGTCGCGACGACGATCTGTTCGATCATTACGCTGCGGTGGCGCAGAGAATCGGAGTT

337 Y T A T D Y A G I L E F L L R R W E V E

1561 TATACCGCGACGGATTACGCAGGGATTTTGGAGTTTTTGTTGCGGCGGTGGGAGGTGGAG

357 K L G M G L S G E G R R A Q D Y L C T L

1621 AAGTTAGGGATGGGTTTGTCCGGCGAAGGAAGGAGAGCACAGGATTATCTGTGTACCTTG

377 P Q R I R R L E E R A N D R V K L A S K

1681 CCGCAGAGGATCAGGAGGTTAGAGGAAAGAGCTAACGATAGAGTCAAACTTGCGTCAAAG

397 S K P S V S F S W I Y G R E V E L *

1741 TCAAAACCTTCTGTTTCCTTCAGCTGGATTTATGGGAGAGAAGTTGAACTA**TAA**

(J) *AtAAD3*

1 M A M A M D R I V F S P S S Y V Y R P C

1 ATGGCGATGGCTATGGATCGGATCGTCTTCTCCCCTTCATCTTACGTCTATCGTCCTTGT

21 Q A R G S R S S R V S M A S T I R S A T

61 CAAGCTAGGGGATCCAGATCTTCCAGAGTTTCCATGGCTTCTACGATTCGCTCTGCGACT
41 T
121 ACgtgagttttaatctctctatttccaattcgtgagcttatttttctttaatgctttgaa
181 tttgttctgatctggagtttggtttgagtatcgatgataaacctctgggctttggttgat
241 ccagtttcttattttcatcaatttgattcccaatgcactctcattgtgtgtagttacgag
301 ttatatgatcacatgtattagtttctggtttgtgaaatgaggaatttgcttcatttgtgt
361 ttaaaaaatctttcatttttactggttttctgatttatctgggagatttctttagatcaa
421 agaatcttgatttttttatttttatttatttgaaaagctatccttacatgcaaaggac
481 aatctttgtctctatgttaaattttgataatctcttcaactttcccaaacgcatgcatga
541 attgttttgtgtttatgactttctttttacttggttgttgacccataagactactatgct
601 aaatcattacttcgttcatttattctcttgtttgattattatgttaagaaagcttgttgg
42 E V T
661 tgtagtagctgcttagactttttctaaagctttatttggttttaattacagAGAGGTTAC
45 N G R K L Y I P P R E V H V Q V K H S M
721 GAATGGAAGGAAACTGTATATCCCTCCCAGAGAGGTGCATGTCCAAGTCAAACATTCAAT
65 P P Q K L E I F K S L E G W A D E T L L
781 GCCGCCACAAAAGTTAGAGATCTTTAAGTCTTTAGAAGGATGGGCTGATGAAACCCTGTT
85 T Y L K P V E K S W Q P T D F L P E P E
841 GACTTATTTAAAACCTGTTGAGAAATCGTGGCAGCCTACCGATTTTCTTCCCGAACCTGA
105 S E G F Y D Q V K E L R E R C K E L P D
901 GTCGGAAGGATTCTATGACCAAGTCAAGGAGCTAAGGGAAAGGTGCAAGGAACTTCCTGA
125 D Y F V V L V G D M I T E E A L P T Y Q
961 TGACTATTTTGTGGTGCTTGTTGGGGATATGATCACTGAAGAAGCACTTCCTACTTACCA
145 T M L N T L D G V R D E T G A S P T P W
1021 GACCATGTTGAACACATTGGATGGTGTTAGGGATGAGACAGGAGCTAGTCCTACTCCTTG
165 A I W T R A W T A E E N R H G D L L N K
1081 GGCTATATGGACCAGGGCTTGGACTGCTGAGGAGAATAGACATGGGGATCTCCTTAACAA
185 Y L Y L S G R V D M R Q I E K T I Q Y L
1141 GTATCTTTATCTGTCTGGTCGGGTAGACATGAGGCAGATTGAAAAGACTATTCAGTACCT
205 I G S G M
1201 GATTGGTTCTGGAATGgtaagctctgctaactttctttttttgctttaacccataatctcc
1261 taaagttgcatttgcgctttaggaagtgactggggatggggatacaatcgttacatgctt
1321 gtttgaattgattgttaaaattccataggtaggggaacagaagaggaataagttggaacc
1381 atttatcagttttactttttttggaacttatcttgtcatttcttagtatggcttatattga
1441 cttcacaatgactatctgcaaatcttcatgttagcaacatacgcatttttttatgtttcaa
1501 gttctgaagatagaattggagtgctgcttagctgccacattaatcactttacggatttcc
1561 attgtgtaaagtttgtattcctaatattcaatgtaagattgatcgatttgtcttatgtcc
210 D P K T E N N P Y L G F I Y T S F Q E
1621 ttagGATCCAAAAACTGAAAACAACCCTTACTTGGGTTTCATCTACACATCATTTCAAGA
229 R A T F I S H G N T A R L A K D R G D L
1681 AAGAGCGACCTTCATCTCCCATGGAAACACTGCCAGACTGGCGAAAGATCGTGGAGATTT
249 K L A Q I C G T I A A D E R R H E T A Y
1741 GAAACTTGCGCAGATATGCGGGACCATTGCTGCTGATGAGAGGCGTCATGAGACTGCTTA
269 T K I V E K L F E I D P D G T I L G L A

1801 CACCAAGATTGTAGAGAAGCTCTTTGAAATTGACCCTGATGGCACGATCTTGGGCCTGGC

289 D M M K K K I S M P A H L M Y D G Q D D

1861 TGATATGATGAAGAAAAAGATATCAATGCCTGCACATTTAATGTATGATGGCCAAGATGA

309 N L F E H F S T V A Q R L G V Y T A K D

1921 TAACCTGTTTGAGCACTTCTCAACCGTTGCCCAGAGGCTCGGTGTCTACACTGCCAAGGA

329 Y A D I L E F L V E R W N V E T L T D L

1981 CTATGCTGATATTCTGGAGTTTCTTGTTGAACGGTGGAATGTGGAGACTTTGACAGACCT

349 S S E G H R A Q

2041 TTCTAGTGAAGGACACAGGGCCCAGgtaaattacctccatgaaacttcttgtgatgaaac

2101 ctccaagtttatgaatacttataacaatcttgtagtagtttttggtcatgaatactatat

2161 tagatatactgttttgtgtttttgatgatctgatcgggacctaaacttgaattctgactc

357 D F V C G L P A R I R K I E

2221 tatcatttcctttgacagGACTTTGTCTGCGGACTACCTGCAAGAATCCGTAAAATTGAA

371 E R A Q G R A K E A A K N I P F S W I F

2281 GAGAGAGCTCAAGGAAGAGCCAAAGAAGCTGCCAAAAACATACCATTCAGCTGGATATTT

391 G R N I R A *

2341 GGTCGAAATATCAGGGCT**TAA**

(**K**) *AtAAD4*

1 M T M M A L L L N S T M T V A M K Q N P

1 **ATG**ACGATGATGGCTCTGCTCTTGAACTCGACGATGACTGTGGCTATGAAGCAGAATCCC

21 A T A V S F M Q T T C L G S S F S P P R

61 GCGACCGCGGTATCTTTTATGCAGACTACTTGTTTGGGATCTAGTTTTTCTCCTCCTCGT

41 H L Q V S C V A T N P S

121 CATCTTCAAGTTTCGTGTGTTGCCACTAACCCGAGgcgagtttttttctttttagatctt

181 caattcttcgcgttgatctcaatgtattgttaataactttttcactcattgggttttata

241 tacttgcatattaacatatgttctataaattacaataagacacgaacctgatcaaaataa

53 K T F R P I K E V

301 cagtgttcttaattaacgatgaaaatgttacagCAAGACGTTTCGACCCATCAAAGAAGT

62 S N Q V T H T I T Q E K L E I F K S M E

361 ATCAAACCAAGTGACACACACAATAACACAAGAGAAACTGGAGATCTTCAAATCAATGGA

82 N W A Q E N L L S Y L K P V E T S W Q P

421 GAATTGGGCCCAAGAAAACTTATTATCTTACCTCAAACCCGTCGAAACTTCATGGCAACC

102 Q D F L P E T K D E D R F Y E Q V K E L

481 ACAAGACTTTTTGCCAGAAACCAAAGACGAAGACCGATTCTACGAACAAGTGAAAGAGCT

122 R D R T K E I P D D Y F V V L V G D M I

541 AAGAGATCGAACAAAAGAGATCCCTGACGATTACTTTGTAGTTCTTGTCGGAGATATGAT

142 T E E A L P T Y Q T V M N T L D G A K D

601 CACCGAAGAAGCATTACCGACTTATCAAACGGTTATGAACACTCTCGACGGAGCCAAAGA

162 E T G V S L T P W A V W L R A W T A E E

661 CGAGACCGGTGTTAGTCTAACACCGTGGGCGGTTTGGCTTAGAGCATGGACGGCGGAGGA

182 N R H G D L L N K Y L Y L S G R V D T R

721 AAATCGACACGGTGATTTGTTGAATAAGTACCTTTACTTATCAGGTCGTGTTGATACGCG

202 H V E K T I Q Y L I G S G M

781 ACATGTTGAAAAGACTATTCAATATCTTATCGGATCCGGCATGgtacgttctttttggta
841 ttggccaaattgttacactcccataaatataggtgtgctcaaaatgtaaattataatttc
216 D T K Y E N N P
901 tttgggaccatattgacaagtttataattatttttcagGACACCAAATATGAGAACAATC
224 Y N G Y I Y T S F Q E R A T F I S H A N
961 CCTACAACGGCTACATCTACACTTCTTTTCAAGAGAGAGCAACTTTTATCTCCCACGCCA
244 T A K L A T T Y G D T T L A K I C G T I
1021 ACACGGCGAAGCTAGCCACGACATACGGCGACACAACTCTCGCCAAAATCTGTGGAACAA
264 A A D E K R H E M A Y T R I V E K L F E
1081 TCGCGGCGGATGAGAAGCGGCACGAGATGGCGTATACGCGGATCGTCGAGAAGCTATTCG
284 I D P D G T V Q A L A S M M R K R I T M
1141 AGATTGATCCCGATGGTACCGTACAAGCTCTAGCGAGTATGATGAGGAAGCGAATCACGA
304 P A Q L M H D G R D D N L F D H Y A A V
1201 TGCCGGCTCAGCTGATGCACGACGGTCGCGATGACAATCTGTTCGATCATTACGCTGCTG
324 A Q R I G V Y T A T D Y A G I L E F L L
1261 TGGCGCAGAGAATCGGAGTGTATACCGCGACGGATTACGCAGGGATTTTGGAGTTTTTGT
344 R R W E V E K L G M G L S G E G R R A Q
1321 TGCGGCGGTGGGAGGTGGAGAAGTTAGGGATGGGTTTGTCCGGTGAAGGAAGGAGAGCAC
364 D Y L C T L P Q R I R R L E E R A D D R
1381 AGGATTATCTGTGTACCTTGCCGCAGAGGATCAGGAGGTTAGAGGAAAGAGCTGACGATA
384 V K R A S K S K P S V S F S W I Y G R E
1441 GAGTCAAACGTGCGTCAAAGTCTAAACCTTCTGTTTCGTTCAGCTGGATTTACGGAGAG
404 V E L *
1501 AAGTTGAACTC**TAA**

(**L**) *AtAAD5*

1 M S M A L L L T S P A M K Q K P A V I T
1 **ATG**TCGATGGCTTTGCTTTTGACATCGCCGGCTATGAAGCAGAAGCCGGCGGTGATAACT
21 S P R R G S S P S R R L R V S C V T T N
61 TCTCCTCGCCGTGGTTCTTCTCCTTCTCGTCGTCTTCGAGTTTCTTGTGTTACCACAAAC
41 P A R
121 CCTGCTAGgtaacgatttattattcgctttctttttaattatgttagatcttcgatttca
181 tttctatagtttttttttttttttttggtagtagagatgtatatgaatttgcgttttcgt
241 tgtcttctatttatcatgtgattatgcgggatctaaatcgttaatccgtcgttttcttaa
301 cacatatagattatttaaatctatttcgctcgcagttactttaaagtttttttactggct
361 attagtttaacgatttatgtcatttttttatttgcaagattttgttgattcacgagtctt
421 aatgtttattaacatgatgagcattcattagtgctatgtccaatgagactataatactac
481 tttcttaaagacattggattccatgtatcttcgttaatcccattattcatattcgtatca
541 tgatataaactttttttttggtccacctgaaaacttttctatattgcagaatcactacac
601 tttttttatgtacactttatgagtgtgtatgcataagttggtgattggtttcacacggat
661 catgacattagctaggtgaccttaagatcctatatagagagataagtttggttgattccc
721 ttatacagatattatttgagtaaactttaatcattttatgcttgaacttaaaatcgttta
781 atttgtttctttttctttttgtttttctgtttttgggttcatactgagacataaaatcgt
841 ttatttaagtaaactttttcttgtcagacaaagtacgattctacttttaaaggctgggt

901 tgtttttcacaattttatttttaccccaccatgcaaaattattgaacaaacgtacgttgt
961 tgttttttttgtttgtatttggtaaaatattgtacatacgttagttgacatattaaaatca
1021 atatacacatcaacaacatccaaaattccaatctggttatggtgacacaacgtcttcaaa
1081 taaaaacgatgggactaatagaccaatcaatatcatcacaattttatatatataaaatga
1141 atacatttcataattctttaaataaaactatagaaatacaacggagttgtttttttaagg
1201 gagtttatgttaaaattacatgttgtgaaagaatacgtatatataaccaccacatttata
1261 gagtttactgtaattgtactattattaacccttttttatagttttgtactataaaaagata
1321 tcgcaaatctttttttaacacgtatccatgactaatatctgtttggtattaaaatttgttc
44 K K N E T C N H F R P I K E
1381 acctcttttttttgtagGAAGAAAAACGAAACATGCAATCATTTTCGACCTATCAAAGAA
58 V N N Q L T H T I P Q E K L E I F K S M
1441 GTAAATAACCAATTAACACACACAATACCACAGGAGAAGCTTGAGATCTTCAAATCAATG
78 E N W A E Q K L L P Y L K P V E D S W Q
1501 GAAAATTGGGCAGAACAAAAACTACTACCTTATCTCAAACCGGTAGAAGATTCATGGCAA
98 P Q D F L P A P E N D D E F Y D R V K E
1561 CCACAAGACTTCTTACCTGCGCCAGAGAACGACGACGAATTCTACGACCGAGTAAAAGAG
118 I R E R T K E I P D D Y F V V L V G D M
1621 ATCAGAGAAAGAACAAAAGAGATACCTGACGATTACTTTGTAGTTCTTGTGGGAGATATG
138 I T E E A L P T Y Q T T L N T L D G V K
1681 ATCACAGAAGAAGCACTTCCAACGTATCAAACGACGTTAAACACACTAGACGGTGTTAAG
158 D E T G G S L S P W A V W I R A W T A E
1741 GATGAAACCGGTGGGAGTTTATCGCCGTGGGCTGTGTGGATTAGAGCCTGGACGGCGGAG
178 E N R H G D L L N K Y L Y L T G R V D M
1801 GAAAACCGTCACGGTGACTTACTCAACAAGTATCTTTATCTAACTGGTCGTGTTGATATG
198 R H V E K T I Q Y L I G S G M
1861 CGACATGTTGAGAAGACTATACAATATCTTATTGGTTCTGGTATGgtaagtatttcacat
1921 ttatcgttactattgatttggttacattattaatattttttttggcaacgttacgtttta
213 D S K F E N N P Y N G F I Y T S F Q E R
1981 gGATTCGAAATTTGAGAACAATCCATACAATGGATTCATCTACACATCATTCCAAGAGCG
233 A T F I S H G N T A R L A T T Y G D V T
2041 AGCAACATTCATCTCTCACGGCAACACGGCTAGGCTAGCCACAACATACGGCGATGTTAC
253 L A K I C G T I A A D E K R H E T A Y T
2101 CCTCGCGAAGATCTGCGGCACAATCGCTGCCGACGAGAAGCGACACGAGACGGCGTACAC
273 K I V E K L F E I D P D G S V Q A L A S
2161 GAAGATAGTGGAGAAGCTATTCGAGATCGACCCTGACGGATCTGTTCAGGCGTTAGCGAG
293 M M K K R I T M P A H L M H D G R D N D
2221 TATGATGAAGAAACGGATCACAATGCCGGCTCATCTTATGCACGACGGAAGAGATAACGA
313 L F D H Y A A V A Q R I G V Y T A A D Y
2281 TTTGTTTGATCATTACGCCGCCGTGGCGCAGCGGATTGGAGTTTACACGGCGGCGGATTA
333 A G I L E F L L R R W K V E S L G L G L
2341 CGCTGGGATACTTGAGTTTCTGTTGCGGCGGTGGAAGGTGGAGAGTTTGGGATTGGGGTT
353 S G E G R R A Q E Y L C T L P Q R I K R
2401 ATCAGGAGAAGGAAGGAGAGCACAGGAGTACTTGTGCACCTTGCCGCAGAGGATCAAGAG
373 L E E R A N D R V K L V S K P S V S F S

2461 GTTAGAGGAAAGAGCTAATGATAGGGTCAAACTTGTGTCAAAACCTTCTGTTTCGTTTAG
393 W V F G R D V K L *
2521 CTGGGTTTTTGGTAGAGATGTGAAACTA**TAG**

(M) *AtAAD6*

1 M L A H K S L L S F T T Q W A T L M P S
1 **ATG**CTTGCGCACAAGTCTCTTCTCTCCTTTACCACCCAATGGGCTACACTAATGCCATCT
21 P S T F L A S R P R G P A K I S A V A A
61 CCGTCTACTTTTCTCGCCTCTCGCCCCCGTGGACCGGCCAAGATCTCGGCCGTGGCAGCA
41 P V R P A L K H Q N K I H T M P P E K M
121 CCAGTGAGGCCGGCTCTAAAACACCAAAACAAAATCCACACCATGCCACCGGAGAAAATG
61 E I F K S L D G W A K D Q I L P L L K P
181 GAGATATTCAAATCTTTAGATGGATGGGCCAAGGATCAAATCTTGCCTCTTCTCAAACCC
81 V D Q C W Q P A S F L P D P A L P F S E
241 GTTGACCAATGTTGGCAACCCGCTTCTTTCTTACCCGACCCGGCCTTACCCTTCTCCGAG
101 F T D Q
301 TTTACCGACCAGgtacgttatatacatcatgaatcttgttattctcttatttatgaagaa
361 gtagaagttattacaaagtttataactttgtcatgaaatgtataaaccagaatacctata
421 ctatataatatgcacattaaaccaagactaaccaataaatgaaaagatgttaaaatgtgt
105 V R E L R E R T A S L P D E Y F
481 atttataatgatagGTTCGTGAGCTGAGGGAAAGAACGGCCTCGCTGCCAGACGAATACT
121 V V L V G D M I T E D A L P T Y Q T M I
541 TCGTGGTGTTGGTTGGAGATATGATAACGGAGGACGCGTTGCCTACTTACCAGACGATGA
141 N T L D G V R D E T G A S E S A W A S W
601 TCAACACCCTTGATGGCGTAAGGGACGAGACTGGTGCGAGTGAGAGCGCGTGGGCAAGTT
161 T R A W T A E E N R H G D L L R T Y L Y
661 GGACACGAGCGTGGACGGCTGAGGAGAACCGTCATGGTGATTTGTTGCGGACTTACTTGT
181 L S G R V D M L M V E R T V Q H L I G S
721 ACTTATCCGGTCGTGTTGATATGCTTATGGTTGAACGCACCGTTCAGCATCTCATCGGCT
201 G M
781 CGGGCATGgtcagttctctttctatacctttatgtcatgatgtaaaaatgcttatactag
841 tttcattagtttagtagttttctgatttatttatttgtttgtgtcattttaatttttttt
901 tatttggtttggttaagttattttttttgttatgcatgtgtggaaacgtaaacataacca
961 aaactatttgccattttgtttgttttgatctggtatgataaaattttaatttggtttttg
1021 gtttgaatacattttggacgatttaaaaagaaaaaaaaatccaccttttaataataataa
1081 aaattaataatatattatgtaagatatatagaaaataaattctgatttttttgtttgttt
1141 agttcgcttacaataacataagctttgggtttaaatgtttttaaataatagaacgaattg
1201 atcacaaacaatttcaatctaagtattttagtagtttacgtttggttggtccggattagg
203 D
1261 tttggttggttcgggttaggtttataccattcataacttttcatattggcaacttcagGA
204 P G T E N N P Y L G F V Y T S F Q E R A
1321 TCCAGGAACTGAGAACAATCCATACTTAGGTTTCGTGTACACGTCATTCCAAGAGCGAGC
224 T F V S H G N T A R L A K S A G D P V L
1381 CACATTTGTGTCTCACGGCAACACGGCAAGGCTAGCCAAGTCCGCAGGAGATCCTGTCCT

244 A R I C G T I A A D E K R H E N A Y V R
1441 CGCTCGCATCTGCGGAACCATTGCAGCTGACGAGAAGCGCCATGAAAACGCTTACGTACG
264 I V E K L L E I D P N G A V S A V A D M
1501 CATCGTTGAGAAGCTCCTCGAGATCGACCCTAACGGTGCAGTCTCAGCCGTGGCCGACAT
284 M R K K I T M P A H L M T D G R D P M L
1561 GATGCGGAAGAAGATCACAATGCCGGCTCATCTAATGACAGACGGTCGAGACCCGATGCT
304 F E H F S A V A Q R L E V Y T A D D Y A
1621 ATTCGAACATTTCTCCGCCGTGGCTCAGCGGCTAGAGGTTTACACGGCGGATGATTACGC
324 D I L E F L V G R W R L E K L E G L T G
1681 TGACATCTTGGAGTTTTTGGTTGGACGGTGGAGATTGGAGAAGCTAGAAGGATTGACGGG
344 E G Q R A Q E F V C G L A Q R I R R L Q
1741 TGAGGGCCAACGTGCACAGGAGTTTGTGTGTGGGTTAGCTCAGAGGATTAGACGCCTTCA
364 E R A D E R A K K L K K T H E V C F S W
1801 AGAGCGTGCAGACGAGAGAGCTAAGAAGCTTAAGAAGACCCATGAGGTTTGCTTTAGTTG
384 I F D K Q I S V *
1861 GATCTTCGATAAGCAGATTAGTGTG**TAA**
