# Supplementary material for: Insights into the Stearoyl-Acyl Carrier Protein Desaturase (SAD) Family in Tigernut (Cyperus esculentus L.), an Oil-Bearing Tuber Plant
Source: Plants (Basel). 2025 Feb 14;14(4):584. doi: 10.3390/plants14040584 (PMC11859870; doi:10.3390/plants14040584)
Supplement: Supplementary file 1 [file plants-14-00584-s001.zip › Figure S2.pdf]

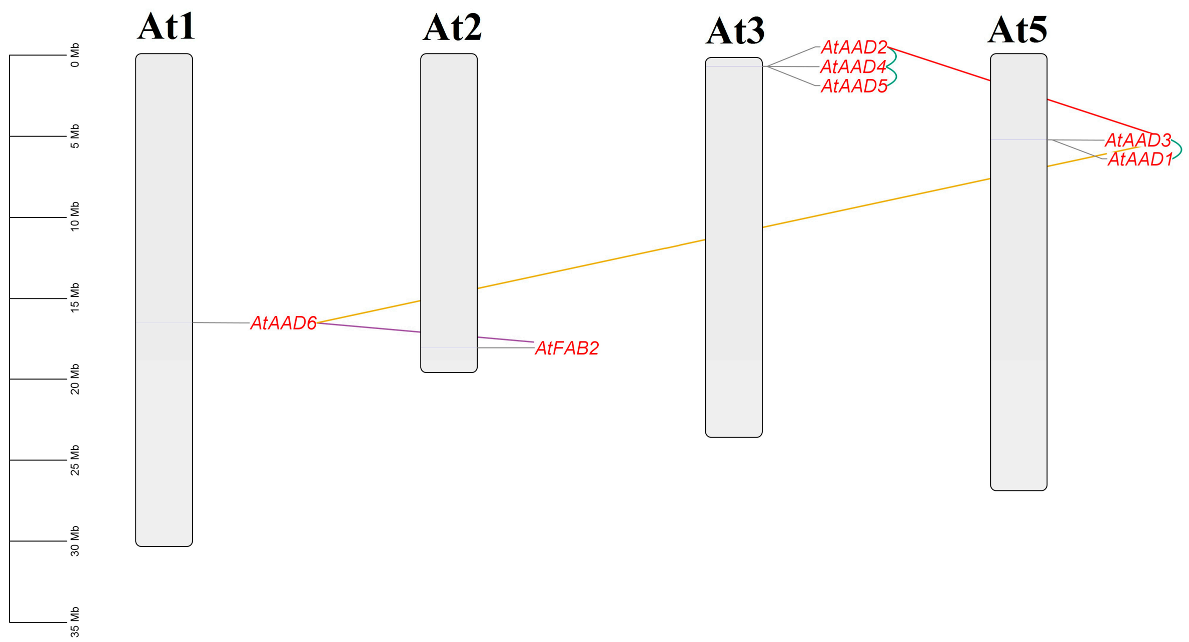

Figure S2: Chromosomal localization and duplication events of *AtSAD* family genes, where WGD, tandem, dispersed, and transposed repeats are connected using red, blue, gold, and purple lines, respectively (AAD: acyl-ACP desaturase; At: *A. thaliana*; FAB2: fatty acid biosynthesis 2; Mb: Megabase; SAD: stearoyl-ACP desaturase).
