# Supplementary material for: Insights into the Stearoyl-Acyl Carrier Protein Desaturase (SAD) Family in Tigernut (Cyperus esculentus L.), an Oil-Bearing Tuber Plant
Source: Plants (Basel). 2025 Feb 14;14(4):584. doi: 10.3390/plants14040584 (PMC11859870; doi:10.3390/plants14040584)
Supplement: Supplementary file 1 [file plants-14-00584-s001.zip › Figure S3.pdf]

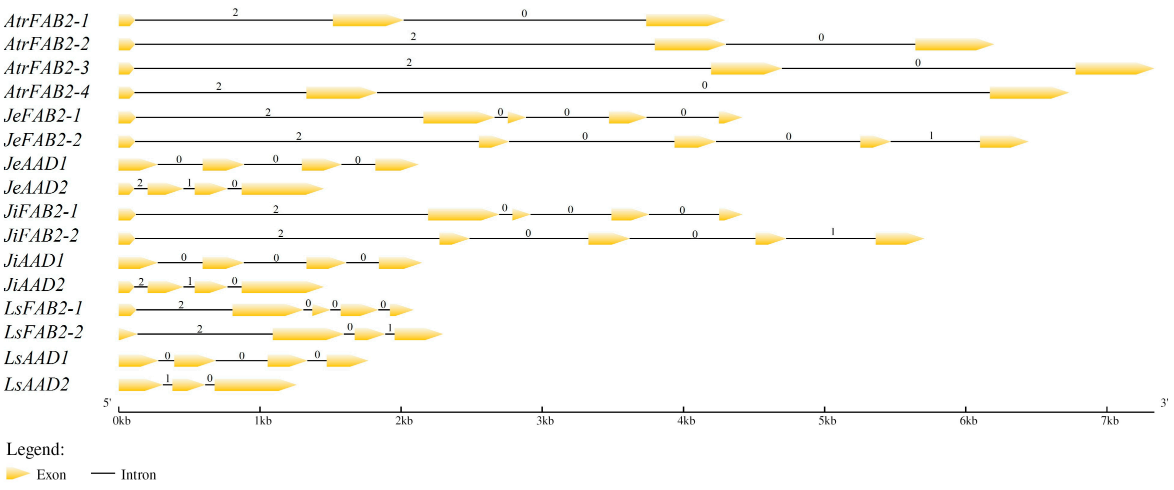

Figure S3: Gene structures of *SAD* family genes in *A. trichopoda*, *J. effuses*, *J. inflexus*, and *L. sylvatica* (AAD: acyl-ACP desaturase; Atr: *A. trichopoda*; CDS: coding sequence; FAB2: fatty acid biosynthesis 2; Je: *J. effusus*; Ji: *J. inflexus*; kb: kilobase; Ls: *L. sylvatica*; SAD: stearoyl-ACP desaturase).
